# Supplementary material for: Amphetamine-like Deferiprone and Clioquinol Derivatives as Iron Chelating Agents
Source: Molecules. 2024 Sep 5;29(17):4213. doi: 10.3390/molecules29174213 (PMC11397356; doi:10.3390/molecules29174213)
Supplement: Supplementary file 1 [file molecules-29-04213-s001.zip › molecules-3120714-supplementary.pdf]

Amphetamine-like Deferiprone and Clioquinol Derivatives as Iron Chelating Agents

Mahmoud El Safadi [1,2], Katie A. Wilson [3,4], Indigo J. Strudwicke [3], Megan L. O'Mara [3,5], Mohan Bhadbhade [6], Tristan Rawling [1] and Andrew M. McDonagh [1,*]

1 School of Mathematical and Physical Sciences, Faculty of Science, University of Technology Sydney, Sydney, NSW 2007, Australia; mahmoudelsafadi@uaeu.ac.ae (M.E.S.); tristan.rawling@uts.edu.au (T.R.)
2 Department of Chemistry, College of Science, United Arab Emirates University, P.O. Box 15551 Al Ain, United Arab Emirates
3 Research School of Chemistry, The Australian National University, Canberra, ACT 2601, Australia; k.wilson@mun.ca (K.A.W.); indigo.strudwicke@anu.edu.au (I.J.S.); m.omara@uq.edu.au (M.L.O.)
4 Department of Biochemistry, Memorial University of Newfoundland, St. John's, NL A1C 5S7, Canada
5 Australian Institute for Bioengineering and Nanotechnology, The University of Queensland, St. Lucia, Qld 4067, Australia
6 Mark Wainwright Analytical Centre, The University of New South Wales, Sydney, New South Wales 2052, Australia; m.bhadbhade@unsw.edu.au
* Correspondence: andrew.mcdonagh@uts.edu.au

General

Reagents and analytical grade solvents were purchased from commercial sources. Dichloromethane, triethylamine and acetonitrile were dried by refluxing over calcium hydride for several hours. Toluene was dried by standing over sodium wire for two days. ^{1}H and ^{13}C NMR spectra were recorded using an Agilent 500 MHz spectrometer (499.86 MHz 1H, 125.70 MHz 13C) using deuterated chloroform ($CDCl_3$) as solvent unless otherwise specified. Low-resolution mass spectra were obtained on an Agilent 6890GC fitted with 5% polysilphenylene, 95% polydimethylsiloxane column, and an Agilent 5973n MS spectrometer. High-resolution mass spectra were obtained on an Agilent 6510 Accurate Mass Q-TOF Mass Spectrometer, equipped with an ESI source. Compounds were purified by column chromatography using either neutral alumina or silica gel (40 – 63 m).

Suitable single crystals were selected for crystallographic studies using a polarizing microscope (Leica M165Z), mounted on a MicroMount (MiTeGen, USA) consisting of a thin polymer tip with a wicking aperture. The X-ray diffraction measurements were carried out on a Bruker kappa-II CCD diffractometer at 150 K by using IμS Incoatec Microfocus Source with Mo-Kα radiation (λ = 0.710723 Å). The single crystal, mounted on the goniometer using cryo loops for intensity measurements, was coated with paraffin oil and then quickly transferred to the cold stream using an Oxford Cryo stream attachment. Symmetry related absorption corrections using the program SADABS (Bruker, 2001) were applied and the data were corrected for Lorentz and polarisation effects using Bruker APEX2 software(Bruker, 2007). The structure was solved by direct methods, and the full-matrix least-square refinement was carried out using Shelxl (Sheldrick, 2008) in Olex2(OLEX, 2009). The non-hydrogen atoms were refined anisotropically. The molecular graphic was generated using program Olex2 (OLEX, 2009).

Synthesis

3-(benzyloxy)-2-methyl-4H-pyran-4-one

Sodium hydroxide (2.00 ml, 7.00 M) was added with stirring to a solution of 2-methyl-3-hydroxy-4-pyrone (1.78 g, 14.0 mmol) in methanol (18 ml), followed by the addition of benzyl bromide (1.91 mL, 16 mmol). The mixture was stirred at reflux for 6 hours. The solvent was then removed to afford an orange oil. The crude compound was dissolved in dichloromethane (20 ml) and transferred to a 100 ml separating funnel. The organic phase washed five times with sodium hydroxide (30ml, 5%). The organic phase was separated then washed with brine (30ml), dried using anhydrous sodium sulfate, filtered, and the solvent removed using a rotary evaporator to give a light yellow oil (2.57 g, 11.9 mmol). Yield 85%; ^{1}H NMR (δ, 500 MHz, $CDCl_3$) 7.60 (d, *J*= 5.5, 1H, H2), 7.41-7.34(m, 5H, H11,12,13,14,15), 6.37(d, *J*= 6.0, 1H, H3), 5.16(s, 2H, H9), 2.09(s, 3H, H16).

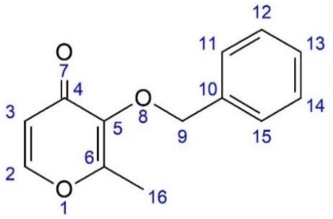

R/*S*-*tert*-Butyl{1-[3-(benzyloxy)-2-methyl-4-oxopyridin-1(4H)-yl]propan-2-yl}carbamate

3-(Benzyloxy)-2-methyl-4H-pyran-4-one (0.170 g, 0.785 mmol) and *rac*-*tert*-butyl(1-aminopropan-2-yl)carbamate (0.150 g, 0.860 mmol) were placed in a single neck round bottom flask and dissolved in ethanol:water (20 mL, 2:3). Sodium hydroxide (2.80 mL, 2.00 M) was added dropwise with stirring at 0 °C. The reaction mixture was stirred at room temperature for 24 h. The volume of the solution was reduced using a rotary evaporator followed by addition of water (20 mL). The mixture was extracted three times with chloroform (20mL). The organic phases were combined and washed three times with brine. The organic phase was separated and dried using sodium sulfate. The solvent was filtered and then evaporated using a rotary evaporator. The crude compound was purified using column chromatography with two solvent systems. First, ethyl acetate: hexane (3:1) was used to elute impurities. Dichloromethane: methanol (9:1) was used to elute the target compound. Removal of the solvent gave a yellow oil (0.100 g, 0.270 mmol). Yield 35%; ^{1}H NMR (δ, 500 MHz, $CDCl_3$) 7.43-7.3 (m, 5H, H18,19,20,21,22), 7.17 (d, 1H, *J*=7.5 Hz, H2), 6.4(d, *J*=7.5Hz, 1H, H3), 5.24(s, 2H, H16), 3.93(m, 1H, H8), 3.82-3.64(m, 2H, H7), 2.18(s, 3H, H23), 1.42(s, 9H, H13,27,26), 1.13(d, *J*=7.0 Hz, 3H, H24), ^{13}C NMR δ: 173, 155, 146, 140, 138, 129, 128, 128, 117, 80.00, 73.0, 58.0, 46.3, 28.3, 18.0, 13.0; HRMS $[M+H]^+$calc. $C_{21}H_{29}N_2O_4$ 373.2127, found 373.2124.

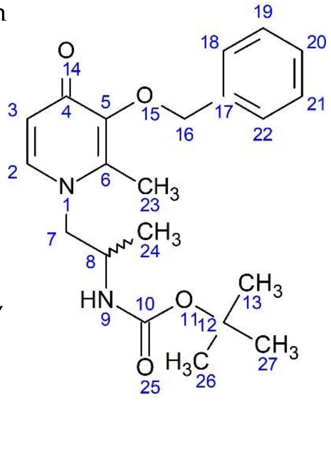

tert-butyl {(2R)-1-[3-(benzyloxy)-2-methyl-4-oxopyridin-1(4H)-yl]propan-2-yl}carbamate

3-(Benzyloxy)-2-methyl-4H-pyran-4-one (0.130 g, 0.601 mmol) and tert-butyl [(2R)-1-aminopropan-2-yl]carbamate (0.130 g, 0.746 mmol) were placed in a single neck round bottom flask and dissolved in ethanol:water (20 mL, 2:3). Sodium hydroxide (2.80 mL, 2.00 M) was added dropwise with stirring at 0 °C. The reaction mixture was stirred at room temperature for 24 h. The volume of the solution was reduced using a rotary evaporator followed by addition of water (20 mL). The mixture was extracted three times with chloroform (20mL). The organic phases were combined and washed three times with brine. The organic phase was separated and dried using sodium sulfate. The solvent was filtered and then evaporated using a rotary evaporator. The crude compound was purified using column chromatography with two solvent systems. First, ethyl acetate: hexane (3:1) was used to elute impurities. Dichloromethane: methanol (9:1) was used to elute the target compound. Removal of the solvent gave a yellow oil (89.5mg, 0.240 mmol). Yield 40%; ^{1}H NMR (δ, 500 MHz, $CDCl_3$) 7.43-7.28 (m, 5H, H18,19,20,21,22), 7.18 (d, 1H, *J*=7.5 Hz, H2), 6.40(d, *J*=7.5Hz, 1H, H3), 5.22(s, 2H, H16), 3.90(m, 1H, H8), 3.84-3.65(m, 2H, H7), 2.17(s, 3H, H23), 1.41(s, 9H, H13,27,26), 1.13(d, *J*=7.0Hz, 3H, H24), ^{13}C NMR δ: 173, 155, 146, 140, 138, 129, 128, 128, 117, 80.00, 73.0, 58.0, 46.3, 28.3, 18.0, 13.0; HRMS $[M+H]^+$calc. $C_{21}H_{29}N_2O_4$ 373.2127, found 373.2128.

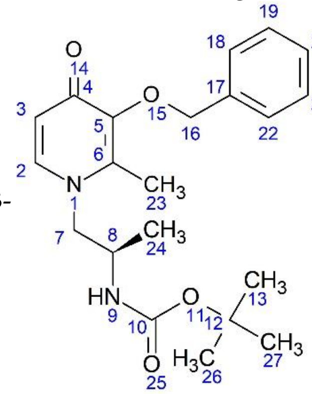

tert-Butyl {(2S)-1-[3-(benzyloxy)-2-methyl-4-oxopyridin-1(4H)-yl]propan-2-yl}carbamate

3-(Benzyloxy)-2-methyl-4H-pyran-4-one (0.200 g, 0.925 mmol) and tert-butyl [(2S)-1-aminopropan-2-yl]carbamate (0.35 0g, 2.00 mmol) were placed in a single neck round bottom flask and dissolved in ethanol:water (20 mL, 2:3). Sodium hydroxide (3.50 mL, 2.00 M) was added dropwise with stirring at 0 °C. The reaction mixture was stirred at room temperature for 24 h. The volume of the solution was reduced using a rotary evaporator followed by addition of water (30 mL). The mixture was extracted three times with chloroform (30mL). The organic phases were combined and washed three times with brine. The organic phase was separated and dried using sodium sulfate. The solvent was filtered and then evaporated using a rotary evaporator. The crude compound was purified using column chromatography with two solvent systems. First, ethyl acetate: hexane (3:1) was used to elute impurities. Dichloromethane: methanol (9:1) was used to elute the target compound. Removal of the solvent gave a yellow oil (141 mg, 0.379 mmol). Yield 41%; ^{1}H NMR (δ, 500 MHz, $CDCl_3$) 7.43-7.28 (m, 5H, H18,19,20,21,22), 7.17 (d, 1H, *J*=7.5 Hz, H2), 6.40(d, *J*=7.5Hz, 1H, H3), 5.24(s, 2H, H16), 3.94(m, 1H, H8), 3.84-3.64(m, 2H, H7), 2.18(s, 3H, H23), 1.41(s, 9H, H13,27,26), 1.13(d, *J*=7.0Hz, 3H, H24), ^{13}C NMR δ: 174, 155, 146, 140, 138, 129, 128, 128, 117, 80.00, 73.0, 58.0, 46.3, 28.3, 18.0, 13.0; HRMS $[M+H]^+$calc. $C_{21}H_{29}N_2O_4$ 373.2127, found 373.2118.

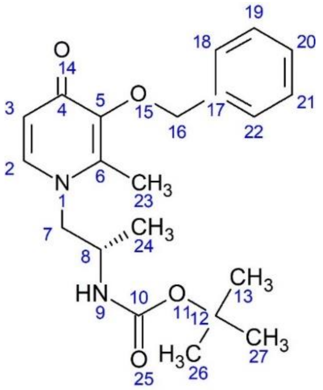

1-(2-Aminoethyl)-3-(benzyloxy)-2-methylpyridin-4(1H)-one

3-(Benzyloxy)-2-methyl-4H-pyran-4-one (0.200 g, 0.925 mmol) and ethane-1,2-diamine (0.615 g, 4.63 mmol) were placed in a single neck round bottom flask and dissolved in ethanol:water (30 mL, 2:3). Sodium hydroxide (16.0 mL, 2.00 M) was added dropwise with stirring at 0 °C. The reaction mixture was stirred at room temperature for 24 h. The volume of the solution was reduced using a rotary evaporator followed by addition of water (30 mL). The mixture was extracted three times with chloroform (30mL). The organic phases were combined and washed three times with brine. The organic phase was separated, dried using sodium sulfate and filtered. The solvent was then evaporated using a rotary evaporator. The crude compound was purified using column chromatography with dichloromethane: methanol: ammonia 83:15:2. Removal of the solvent gave a light brown oil (83.43 mg, 0.323 mmol). Yield 35%; ^{1}H NMR (δ, 500 MHz, $CDCl_3$) 7.42-7.27 (m, 5H, H14, 15, 16, 17, 18), 7.26 (d, 1H, *J*=6.5 Hz, H2), 6.44(d, *J*=7.0 Hz, 1H, H3), 5.23(s, 2H, H12), 3.82(t, *J*=6.0 Hz, 2H, H7), 2.96(t, *J*=6.5Hz, 2H, H8), 2.12(s, 3H, H19), ^{13}C NMR δ: 173, 146, 141, 139, 137, 129, 128, 128, 117, 73.0, 56.0, 42.0, 12.5; HRMS $[M+H]^+$calc. $C_{15}H_{19}N_2O_2$ 259.1447, found 259.1442.

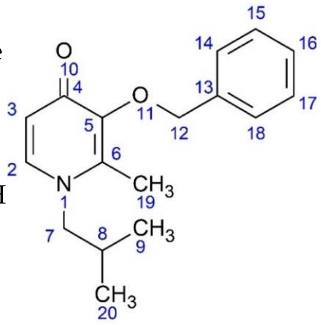

3-(benzyloxy)-2-methyl-1-(2-methylpropyl)pyridin-4(1H)-one

3-(Benzyloxy)-2-methyl-4H-pyran-4-one (0.170 g, 0.785 mmol) and 2-methylpropan-1-amine (78.0 µL, 0.785 mmol) were placed in a single neck round bottom flask and dissolved in ethanol:water (20 mL, 2:3). Sodium hydroxide (2.81 mL, 2.00 M) was added dropwise with stirring at 0 °C. The reaction mixture was stirred at room temperature for 24 h. The volume of the solution was reduced using a rotary evaporator followed by addition of water (30 mL). The mixture was extracted three times with chloroform (30mL). The organic phases were combined and washed three times with brine. The organic phase was separated and dried using sodium sulfate. The solvent was filtered and then evaporated using a rotary evaporator. The crude compound was purified using column chromatography with two solvent systems. First, ethyl acetate: hexane (3:1) was used to elute impurities. Dichloromethane: methanol (9:1) was used to elute the target compound. Removal of the solvent gave an orange oil (198 mg, 0.73 mmol). Yield 92.9%; ^{1}H NMR (δ, 500 MHz, $CDCl_3$) 7.42-7.27 (m, 5H, H14, 15, 16, 17, 18), 7.14 (d, 1H, *J*=7.5 Hz, H2), 6.42(d, *J*=7.5Hz, 1H, H3), 5.25(s, 2H, H12), 3.55(d, *J*=7.5Hz, 2H, H7), 2.07(s, 3H, H19), 1.9(m, 1H, H8), 0.9(d, *J*=6.5Hz, 6H, H9, 20), ^{13}C NMR δ: 173, 146, 139, 138, 129, 128, 128, 117, 72.9, 60.8, 29.7, 19.6, 12.6; HRMS $[M+H]^+$calc. $C_{17}H_{22}NO_2$ 272.1651, found 272.1650.

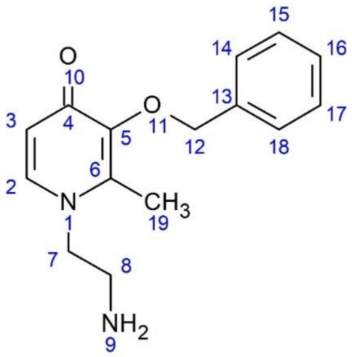

1-(2-ammoniopropyl)-3-(benzyloxy)-4-hydroxy-2-methylpyridinium

tert-Butyl[1-[3-(benzyloxy)-2-methyl-4-oxopyridin-1(4H)-yl]propan-2-yl]carbamate (0.100 g, 0.270 mmol) was dissolved in diethyl ether (7.00mL) and placed in a 50 mL round bottom flask flushed with nitrogen gas and sealed with a septum. Hydrogen chloride in diethyl ether (10.00 mL, 2.00 M) was added to the flask by syringe. After 3 hours, a fine yellow powder had formed. The volatile components were removed using rotary evaporator to give a yellow solid (70.1 mg, 0.256 mmol). Yield 95%; ^{1}H NMR (δ, 500 MHz, DMSO) 8.47 (s, 3H, H20a,b,c), 8.35 (d, 1H, *J*=7.0 Hz, H5), 7.48-7.37(m, 5H, H11,12,13,14,15), 7.20(d, *J*=7.0Hz, 1H, H4), 5.10(s, 2H, H9), 4.52-4.43(m, 2H, H17), 3.65(m, 1H, H18), 2.50(s, 3H, H7), 1.25(d, *J*=6.0Hz, 3H, H19); ^{13}C NMR δ: 144, 143, 137, 129, 129, 129, 114, 74.4, 58.1, 46.4, 16.3, 14.0; HRMS $[M]^{2+}$ calc. $[C_{16}H_{22}N_2O_2]^{2+}$ 273.1609, found 273.1571.

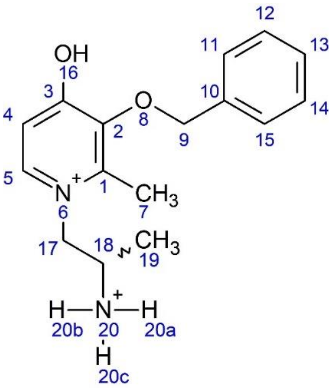

1-[(2*R*)-2-Ammoniopropyl]-3-(benzyloxy)-4-hydroxy-2-methylpyridinium

Tert-butyl [(2R)-1-[3-(benzyloxy)-2-methyl-4-oxopyridin-1(4H)-yl]propan-2-yl]carbamate (89.5 g, 0.240 mmol) was dissolved in diethyl ether (5.00 mL) and placed in a 50 mL round bottom flask flushed with nitrogen gas and sealed with a septum. Hydrogen chloride in diethyl ether (10.00 mL, 2.00 M) was added to the flask by syringe. After 3 hours, a fine yellow powder formed. The volatile components were removed using a rotary evaporator to give a yellow solid (62.5 mg, 0.228 mmol). Yield 95%; ^{1}H NMR (δ, 500 MHz, DMSO) 8.47 (s, 3H, H20a,b,c), 8.35 (d, 1H, *J*=7.0 Hz, H5), 7.48-7.37(m, 5H, H11,12,13,14,15), 7.20(d, *J*=7.0Hz, 1H, H4), 5.10(s, 2H, H9), 4.52-4.43(m, 2H, H17), 3.65(m, 1H, H18), 2.50(s, 3H, H7), 1.25(d, *J*=6.0Hz, 3H, H19); ^{13}C NMR δ: 144 (1 C, C3), 143 (1C, C5), 137(1 C, C10), 129 (2C, C12, 14), 129 (2C, C11,15), 129(1C, C13), 114 (1C, C4), 74.4(1C, C9), 58.1(1C, C18), 46.4(1C, C17), 16.3 (1C, C19), 14 (1C, C7); HRMS $[M]^{2+}$ calc. $[C_{16}H_{22}N_2O_2]^{2+}$ 273.1609, found 273.1574.

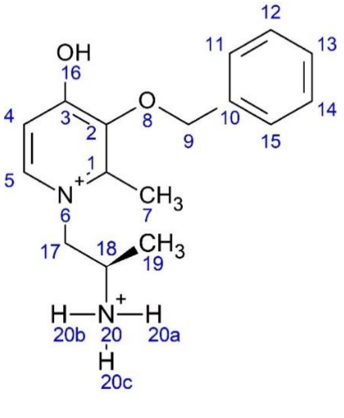

1-[(2*S*)-2-Ammoniopropyl]-3-(benzyloxy)-4-hydroxy-2-methylpyridinium

Tert-butyl [(2S)-1-[3-(benzyloxy)-2-methyl-4-oxopyridin-1(4H)-yl]propan-2-yl]carbamate (141 mg, 0.379 mmol) was dissolved in diethyl ether (10mL) and placed in a 50mL round bottom flask flushed with nitrogen gas and sealed with a septum. Hydrogen chloride in diethyl ether (10.00 mL, 2.00M) was added to the flask by syringe. After 3 hours, a fine yellow powder had formed. The volatile components were removed using rotary evaporator to give a yellow solid (101 mg, 0.367 mmol). Yield 97%; ^{1}H NMR (δ, 500 MHz, DMSO) 8.47 (s, 3H, H20a,b,c), 8.38 (d, 1H, *J*=7.0 Hz, H5), 7.48-7.37(m, 5H, H11,12,13,14,15), 7.20(d, *J*=6.5Hz, 1H, H4), 5.10(s, 2H, H9), 4.57-4.41(m, 2H, H17), 3.66(m, 1H, H18), 2.5(s, 3H, H7), 1.26(d, *J*=6.5Hz, 3H, H19); ^{13}C NMR δ: 144 (1 C, C3), 143 (1C, C5), 137(1 C, C10), 129 (2C, C12, 14), 129 (2C, C11,15), 129(1C, C13), 114 (1C, C4), 74.3(1C, C9), 58.0 (1C, C18), 46.4(1C, C17), 16.3 (1C, C19), 14.0 (1C, C7); HRMS [M]$^{2+}$ calc. [$C_{16}H_{22}N_2O_2$]$^{2+}$ 273.1609, found 273.1574.

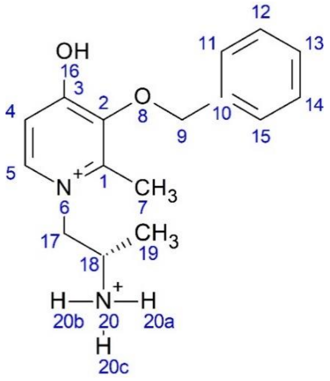

1-(2-Ammoniopropyl)-3,4-dihydroxy-2-methylpyridinium.2HCl

1-(2-Ammoniopropyl)-3-(benzyloxy)-4-hydroxy-2-methylpyridinium (70.1 mg, 0.256 mmol) was dissolved in methanol:water (100 mL, 37%) and placed in a 250 mL quick fit conical flask. Aqueous HCl (10.00 mL, 3.00 M) was added followed by palladium on carbon (0.200 g, 5%). The mixture was stirred under an atmosphere of H_2 for 4 hours. The solution was filtered using a Büchner funnel and the filtrate was evaporated using a rotary evaporator and the resulting materials was placed in a freeze drier for 24 hrs to remove all solvent residue to give a purple solid (44.8 mg, 0.2432 mmol). Yield 95%; Optical rotation +2^0; ^{1}H NMR (δ, 500 MHz, DMSO) 8.52 (s, 3H, H13a,b,c), 8.27 (d, 1H, *J*=7.0 Hz, H5), 7.21(d, *J*=7.0Hz, 1H, H4), 4.60-4.48(m, 2H, H10), 3.68(m, 1H, H11), 2.54(s, 3H, H7), 1.28(d, *J*=6.5Hz, 3H, H12); ^{13}C NMR δ: 160, 144, 143, 139, 111, 58.0, 46.4, 16.3, 13.5; HRMS [M+1]$^{2+}$ calc. [$C_9H_{16}N_2O_2$]$^{2+}$ 183.1139, found 183.1101; FTIR (neat) 3375, 2834, 1635, 1575, 1507, 1328, 1221, 1107, 1029, 910.0, 792.0, 595.0.

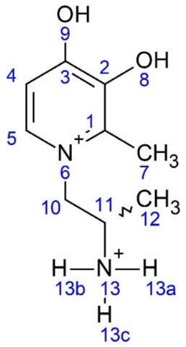

1-[(2*R*)-2-Ammoniopropyl]-3,4-dihydroxy-2-methylpyridinium.2HCl

1-[(2*R*)-2-Ammoniopropyl]-3-(benzyloxy)-4-hydroxy-2-methylpyridinium (62.5 mg, 0.228 mmol) was dissolved in methanol:water (100 mL, 37%) and placed in a 250 mL quick fit conical flask. Aqueous HCl (10.00mL, 3.00M) was added followed by palladium on carbon (0.200 g, 5%). The mixture was stirred under an atmosphere of H_2 for 4 hours. The solution was filtered using a Büchner funnel and the filtrate was evaporated using a rotary evaporator and the resulting materials was placed in a freeze drier for 24 hrs to remove all solvent residue to give a purple solid (40.7 mg, 0.221 mmol). Yield 97%; Optical rotation -22^0; ^{1}H NMR (δ, 500 MHz, DMSO) 8.58 (s, 3H, H13a,b,c), 8.29 (d, 1H, *J*=6.5 Hz, H5), 7.23(d, *J*=6.0Hz, 1H, H4), 4.6-4.49(m, 2H, H10), 3.68(m, 1H, H11), 2.54(s, 3H, H7), 1.29(d, *J*=6.5Hz, 3H, H12); ^{13}C NMR δ: 160 , 144, 143, 139, 111, 58, 46.4, 16.3, 13.5; HRMS [M+1]$^{2+}$ calc. [$C_9H_{16}N_2O_2$]$^{2+}$ 183.1139, found 183.1122, FTIR (neat) 3375, 2835, 1635, 1575, 1507, 1328, 1222, 1107, 1029, 910.00, 792.0, 597.0.

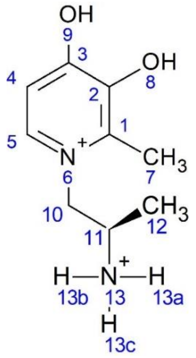

1-[(2*S*)-2-ammoniopropyl]-3,4-dihydroxy-2-methylpyridinium.2HCl

1-[(2S)-2-ammoniopropyl]-3-(benzyloxy)-4-hydroxy-2-methylpyridinium (101 mg, 0.367 mmol) was dissolved in methanol:water (150 mL, 37%) and placed in a 250mL quick fit conical flask. Aqueous HCl (10.00mL, 3.00M) was added followed by palladium on carbon (0.350 g, 5%). The mixture was stirred under an atmosphere of H_2 for 4 hours. The solution was filtered using a Büchner funnel and the filtrate was evaporated using a rotary evaporator and the resulting materials was placed in a freeze drier for 24 hrs to remove all solvent residue to give a purple solid (65.0 mg, 0.352 mmol). Yield 96%; Optical rotation +22^0; ^{1}H NMR (δ, 500 MHz, DMSO) 8.51 (s, 3H, H13a,b,c), 8.28 (d, 1H, *J*=6.5 Hz, H5), 7.22(d, *J*=6.0Hz, 1H, H4), 4.60-4.50(m, 2H, H10), 3.69(m, 1H, H11), 2.54(s, 3H, H7), 1.29(d, *J*=6.5Hz, 3H, H12); ^{13}C NMR δ: 160, 144, 143, 139, 111, 58.0, 46.4, 16.3, 13.5; HRMS [M+1]$^{2+}$ calc. [$C_9H_{16}N_2O_2$]$^{2+}$ 183.1139, found 183.1138. FTIR (neat) 3375, 2834, 1635, 1575, 1507, 1328, 1221, 1107, 1029, 910.00, 792.0, 597.0.

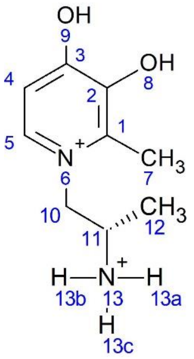

1-(2-Ammonioethyl)-3,4-dihydroxy-2-methylpyridinium.2HCl

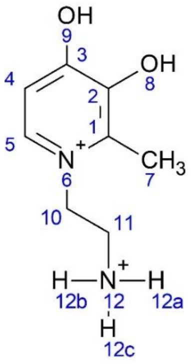

1-(2-aminoethyl)-3-(benzyloxy)-2-methylpyridin-4(1*H*)-one (83.4 mg, 0.323 mmol) was dissolved in methanol:water (120 mL, 37%) and placed in a 250mL quick fit conical flask. Aqueous HCl (10.00mL, 3.00M) was added followed by palladium on carbon (0.300 g, 5%). The mixture was stirred under an atmosphere of H_2 for 4 hours. The solution was filtered using a Büchner funnel and the filtrate was evaporated using a rotary evaporator and the resulting materials was placed in a freeze drier for 24 hrs to remove all solvent residue to give a bright purple Solid (53.87 mg, 0.3165 mmol). Yield 98%; ^{1}H NMR (δ, 500 MHz, CD_3OD) 8.18 (d, 1H, *J*=7.0 Hz, H5), 7.14(d, *J*=7.0Hz, 1H, H4), 4.69(t, 2H, *J*=7.0Hz, H10), 3.47(t, 2H, *J*=7.5Hz, H11), 2.67(s, 3H, H7); ^{13}C NMR δ: 160.0, 149, 144, 138, 111, 52.5, 38.1, 11.5; HRMS $[M+1]^{2+}$ calc. $[C_8H_{14}N_2O_2]^{2+}$ 169.0983, found 169.0947. FTIR (neat) 3160, 2906, 1629, 1461, 1324, 1254, 1152, 1021, 947.0, 818.0, 759.0, 640.0, 601.0.

3,4-Dihydroxy-2-methyl-1-(2-methylpropyl)pyridinium.HCl

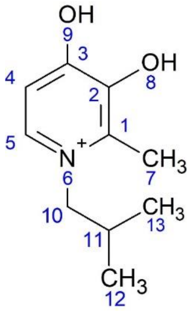

3-(benzyloxy)-2-methyl-1-(2-methylpropyl)pyridin-4(1*H*)-one (198 mg, 0.730 mmol) was dissolved in methanol:water (150 mL, 37%) and placed in a 250mL quick fit conical flask. Aqueous HCl (10.00mL, 3.00M) was added followed by palladium on carbon (0.600 g, 5%). The mixture was stirred under an atmosphere of H_2 for 4 hours. The solution was filtered using a Büchner funnel and the filtrate was evaporated using a rotary evaporator and the resulting materials was placed in a freeze drier for 24 hrs to remove all solvent residue to give a bright purple Solid (125 mg, 0.686 mmol). Yield 94%; ^{1}H NMR (δ, 500 MHz, DMSO) 8.21 (d, 1H, *J*=7.0Hz, H5), 7.36(d, *J*=7.0Hz, 1H, H4), 4.18(d, 2H, *J*=7.5Hz, H10), 2.52(s, 3H, H7), 2.08(m, 1H, H11), 0.88(d, *J*= 6.5Hz, 6H, H12,13); ^{13}C NMR δ: 159, 144, 142, 139, 111, 63.0, 29.0, 19.5, 13.1; HRMS $[M]^+$ calc. $[C_{10}H_{16}NO_2]^+$ 182.1181, found 182.1174. FTIR (neat) 3343, 2962, 1631, 1495, 1333, 1133, 1032, 792, 594.

General procedure for synthesis of iron complexes of 1-5

A methanolic solution of ferric nitrate nonahydrate (1.50 mL, 0.500 mM) was added to a methanolic solution of ligand **1-5** (1.5 mL, 1.5 mM). The solution was stirred at room temperature for 15 min. Crystals were grown by diffusion of diethyl ether into methanolic solution in a sealed vessel.

Fe^{III}(1)$_3$: Maroon coloured crystals. HRMS $[M]^+$ calc. $[C_9H_{13}N_2O_2]_2Fe$ 416.1350, found 416.1319 with % abund. *m/z* 416.1319 (6.36), 417.1316 (1.3), 418.1262 (100), 419.1288(23.67) and $[M+1]^+$ calc. $[C_9H_{13}N_2O_2]_3Fe$ 598.2399, found 598.234 with % abund. *m/z* 598.2340 (6.36), 600.2296 (100), 601.2321(34.31) FTIR (neat) 3367, 1604, 1492, 1348, 1279, 825.0, 577.0; UV (10% MeOH in PBS, ε(M^{-1}, cm^{-1})))) 460(4320), 292(27700).

Fe^{III}(2)$_3$: Maroon coloured crystals. HRMS $[M]^+$ calc. $[C_9H_{13}N_2O_2]_2Fe$ 416.1350, found 416.1331 with % abund. *m/z* 416.1403 (6.36), 417.14 (1.3), 418.1336 (100), 419.1356(23.67) and $[M+1]^+$ calc. $[C_9H_{13}N_2O_2]_3Fe$ 598.2399, found 598.2450 with % abund. *m/z* 598.2450 (6.36), 600.2403 (100), 601.2436(34.31) FTIR (neat) 3367, 1604, 1492, 1348, 1279, 825.0, 577.0; UV (10% MeOH in PBS, ε(M^{-1}, cm^{-1})), 460(4308), 292(28044).

Fe^{III}(3)$_3$: Maroon coloured crystals. HRMS $[M]^+$ calc. $[C_9H_{13}N_2O_2]_2Fe$ 416.1350, found 416.1332 with % abund. *m/z* 416.1332 (6.36), 417.1410 (1.3), 418.1289 (100), 419.1314(23.67), 420.1334(3.8) and $[M+1]^+$ calc. $[C_9H_{13}N_2O_2]_3Fe$ 598.2399, found 598.235 with % abund. *m/z* 598.235 (6.36), 600.2318 (100), 601.2362(34.31) FTIR (neat) 3368, 1604, 1491, 1348, 1279, 825.0, 576.0; UV (10% MeOH in PBS, ε(M^{-1}, cm^{-1})), 463(4156), 290.00(27600).

Fe^{III}(4)$_3$: Maroon coloured crystals. HRMS $[M]^+$ calc. $[C_8H_{11}N_2O_2]_2Fe$ 388.1032, found 388.1014 with % abund. *m/z* 388.1014 (6.36), 389.1020 (1.22), 390.0966 (100), 391.0993(21.46), 392.1008(3.31) and $[M+1]^+$ calc. $[C_8H_{11}N_2O_2]_3Fe$ 556.1930, found 556.1800 with % abund. *m/z* 556.1800(6.35), 558.1821 (100), 559.1845(31.02) FTIR (neat) 3365, 1552, 1489, 1345, 1280, 1043, 823.0, 542.0; UV (10% MeOH in PBS, ε(M^{-1}, cm^{-1})), 462(4150), 290(28000).

Fe^{III}(5)$_3$: Maroon coloured crystals. HRMS [M]$^+$ calc. $[C_{10}H_{14}NO_2]_2Fe$ 414.1439, found 414.1269 with % abund. *m/z* 414.1269 (6.36), 415.1316 (1.45), 416.1224 (100), 417.1251(25.12), 418.1280(4.14) and [M+1] $^+$ calc. $[C_{10}H_{14}NO_2]_3Fe$ 595.2543, found 595.2263 with % abund. *m/z* 595.2263(6.34), 597.2243 (100), 598.2267(36.48), 599.2320(8) FTIR (neat) 3366, 2959, 1725, 1607, 1491, 1343, 1261, 825.0, 561.0; UV (10% MeOH in PBS, ε(M^{-1}, cm^{-1})), 463(4180), 286(28200).

8-Hydroxyquinoline-2-carbaldehyde

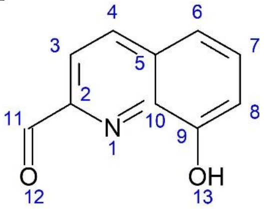

2-methylquinolin-8-ol (2.00 g, 12.5 mmol) and selenium dioxide (1.75g, 15.5mmol) were dissolved in dioxane (150 mL) and water (2mL). The mixture was stirred at 80 ⁰C for 24 hours under nitrogen, cooled to room temperature and filtered. The solvent of the filtrate was evaporated using a rotary evaporator and the crude material was purified using silica gel column chromatography eluting with ethylacetate:n-hexane (5:95). Light yellow solid (1.73 g, 10 mmol). Yield 80%; ^{1}H NMR (δ, 500 MHz, $CDCl_3$) 10.23 (S, 1H, H11), 8.34(d, *J*=8.5Hz, 1H, H4), 8.08(d, *J*=8.5Hz, 1H, H3), 7.63(m, 1H, H7), 7.45(dd, *J*=8.5, 1Hz, 1H, H6), 7.3(dd, *J*=7.5, 1.0 Hz, 1H, H8).

8-Hydroxyquinoline-5-carbaldehyde

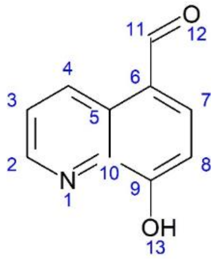

8-Hhydroxyquinoline (30.00 g, 0.210 mol) was dissolved in ethanol (80 mL) and placed in a 250 mL round bottom flask. Aqueous sodium hydroxide (36%) was added until a yellow precipitate started to appear. The mixture was heated with stirring to ~100 ⁰C until the precipitate disappeared. The temperature was decreased to 50 ⁰C and chloroform (50 mL) was added slowly with vigorous stirring. The reaction mixture was stirred at reflux for 12 h. After cooling to room temperature, the solvent was removed using a rotary evaporator. Water (100 mL) was added to the residue and the pH adjusted to 5 by using aqueous hydrochloric acid (1 M). A brown/yellow precipitate formed, which was collected by filtration then dissolved in chloroform. The chloroform mixture was filtered and the solvent removed using a rotary evaporator. The crude product was purified by Soxhlet extraction for three days with petroleum spirit (100-200 ⁰C fraction). The solid material was recrystallised from absolute ethanol to give white crystals (3.3 g, 18.9 mmol). Yield 9%; ^{1}H NMR (δ, 500 MHz, $CDCl_3$) 10.15 (s, 1H, H11), 9.7(dd, *J*=8.5Hz, 1.5Hz, 1H, H2), 8.88(dd, *J*=4.5, 1.5Hz 1H, H4), 8.02(d, *J*=8.0Hz, 1H, H7), 7.68(m, 1H, H3), 7.3(d, *J*=8.0Hz, 1H, H8).

8-Hydroxyquinoline-7-carbaldehyde

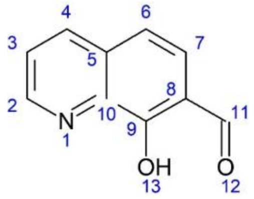

This compound was prepared using the same synthetic method as used for 8-hydroxyquinoline-5-carbaldehyde but the crude product was not subjected to Soxhlet extraction. The crude product was purified from its isomer by column chromatography with a gradient of petroleum spirit: ethyl acetate (20:1) and then crystallised from absolute ethanol to give light yellow crystals (1.50 g, 8.40 mmol). Yield 4%; ^{1}H NMR (δ, 500 MHz, $CDCl_3$) 10.37 (s, 1H, H11), 8.96(dd, *J*=4.0, 2Hz, 1H, H2), 8.18(dd, *J*=8.5, 1.5Hz, 1H, H4), 7.76(d, *J*=8.5Hz, 1H, H6), 7.61(m, 1H, H3), 7.39(d, *J*=9.0 Hz, 1H, H7).

8-(Benzyloxy)quinoline-2-carbaldehyde

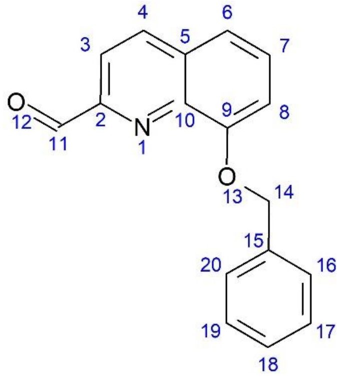

8-Hydroxyquinoline-2-carbaldehyde (0.100 g, 0.570 mmol) was dissolved in methanol (10 mL). Aqueous sodium hydroxide (5 mL, 0.1 M) was added dropwise to the stirred methanolic solution. Benzyl bromide (84.0 µL) was added followed by heating to reflux for 18 hours. After 18 hours the solvent volume was reduced using a rotary evaporator. The crude product was dissolved in dichloromethane (20 ml) and the solution transferred to a separating funnel for liquid-liquid extraction. The organic phase was washed using aqueous sodium hydroxide (20ml, 5%). This process was repeated three times, and the collected organic phase was dried using sodium sulfate. The solution was filtered and the dichloromethane was evaporated using a rotary evaporator to give a yellow solid (1.12 g, 0.430 mmol). Yield 85%; ^{1}H NMR (δ, 500 MHz, $CDCl_3$) 10.3 (s, 1H, H11), 8.30(d, *J*=8.5Hz, 1H, H4), 8.08(d, *J*=8.5Hz, 1H, H3), 7.63-7.16(m, 8H, H6,7,8,16,17,18,19,20), 5.51 (s, 2H, H14).

2-(-2-nitroprop-1-en-1-yl)quinolin-8-ol

8-Hydroxyquinoline-2-carbaldehyde (0.100 g, 0.580 mmol) was added to a single necked round bottom flask and nitroethane (10 ml) and ammonium acetate (0.1 g) were added. The solution was stirred at reflux for 5 hours. The solution was concentrated using a rotary evaporator. The crude product was dissolved in 20mL of dichloromethane and washed three times with brine water. The organic phase was collected and dried using sodium sulfate, filtered, and the solvent was evaporated using a rotary evaporator. The crude compound was purified by silica gel column chromatography with mobile phase dichloromethane: hexane with ratio 75:25. Yellow solid (126.9 mg, 0.550 mmol). Yield 95%; ^{1}H NMR (δ, 500 MHz, $CDCl_3$) 8.26 (d, 1H, *J*=9.0 Hz, H4), 8.14 (s, 1H, H11), 7.58-7.52(m, 2H, H7,3), 7.39(d, 1H, *J*=8.5 Hz, H6), 7.25 (d, 1H, overlapped with $CDCl_3$ peak, H8), 2.82(s, 3H, H13); ^{13}C NMR δ: 137, 130, 129, 125, 118, 111, 14.7.

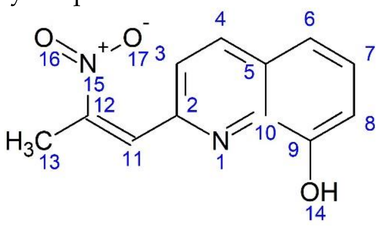

4-(-2-nitroprop-1-en-1-yl)quinolin-8-ol

8-hydroxyquinoline-4-carbaldehyde (0.100 g, 0.580 mmol) was placed in a single neck round bottom flask and nitroethane (10 ml) and ammonium acetate (0.100 g) was added. The solution was stirred at reflux for 5 hours. After 5 hours, the solution was concentrated by using the rotary evaporator. Then the crude product was dissolved in 20mL of dichloromethane and washed three times with brine water. The organic phase was collected and dried from water by using sodium sulfate. Finally, dichloromethane was evaporated by rotary evaporator. The crude compound was purified by silica gel column chromatography with mobile phase dichloromethane: hexane with ratio 75:25 respectively. Yellow solid (124 mg, 0.540 mmol). Yield 93%; ^{1}H NMR (δ, 500 MHz, $CDCl_3$) 8.85 (d, 1H, *J*=4.0 Hz, H2), 8.44 (s, 1H, H11), 7.56(m, 1H, H7), 7.37-7.35(m, 2H, H6,3), 7.26 (d, 1H, overlapped with $CDCl_3$ peak, H8), 2.34(s, 3H, H13); ^{13}C NMR δ: 147, 128.9, 128.8, 121, 114, 111, 14.2.

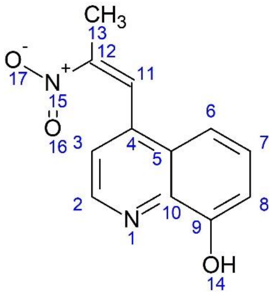

7-(-2-nitroprop-1-en-1-yl)quinolin-8-ol

8-Hydroxyquinoline-7-carbaldehyde (0.100 g, 0.580 mmol) was added to single neck round bottom flask and nitroethane (10 ml) and ammonium acetate (0.100 g) were added. The solution was stirred at reflux for 5 hours and then the solution was concentrated using a rotary evaporator. The crude product was dissolved in 20mL of dichloromethane and washed three times with brine water. The organic phase was collected, dried using sodium sulfate and filtered. The solvent was evaporated using a rotary evaporator. The crude compound was purified by silica gel column chromatography with mobile phase dichloromethane: hexane with ratio 75:25. Yellow solid (124.18 mg, 0.54 mmol). Yield 94%; ^{1}H NMR (δ, 500 MHz, $CDCl_3$) 8.85 (dd, 1H, *J*=1.5, 4Hz, H2), 8.48 (s, 1H, H11), 8.20(dd, 1H, *J*= 8.0, 1.5 Hz, H4), 7.54(m, 1H, H3), 7.51 (d, 1H, *J*=9.0Hz , H6), 7.40 (d, 1H, *J*=9.0Hz, H7), 2.34(s, 3H, H13); ^{13}C NMR δ: 152, 149, 138, 136, 131, 129, 128, 127, 123, 118, 114, 14.6.

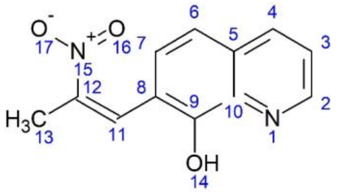

5-(-2-nitroprop-1-en-1-yl)quinolin-8-ol

8-Hydroxyquinoline-7-carbaldehyde (0.100 g, 0.580 mmol) was added to single neck round bottom flask and nitroethane (10 ml) and ammonium acetate (0.100 g) were added. The solution was stirred at reflux for 5 hours and then the solution was concentrated using a rotary evaporator. The crude product was dissolved in 20mL of dichloromethane and washed three times with brine water. The organic phase was collected, dried using sodium sulfate and filtered. The solvent was evaporated using a rotary evaporator. The crude compound was purified by silica gel column chromatography with mobile phase dichloromethane: hexane with ratio 75:25. Yellow solid (124.2 mg, 0.540 mmol). Yield 94%; ^{1}H NMR (δ, 500 MHz, $CDCl_3$) 8.89 (dd, 1H, *J*=1.5, 4.5Hz, H2), 8.58 (s, 1H, H11), 8.33(dd, 1H, *J*=1.5, 8.5Hz H4), 7.6(m, 1H, H3), 7.55 (d, 1H, *J*=8.0Hz , H7), 7.26 (d, 1H, *J*=8.0Hz, H8), 2.44(s, 3H, H13); ^{13}C NMR δ: 154, 148.4, 148.3, 138, 133, 130, 129.9, 127, 123, 120, 110, 14.4.

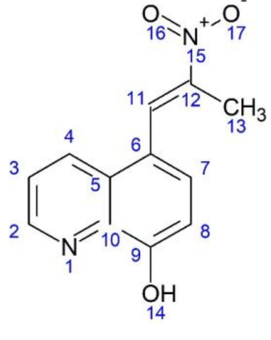

2-(2-aminopropyl)quinolin-8-ol

Anhydrous tetrahydrofuran (10 ml) was transferred into a 100ml three-necked round bottom flask equipped with a magnetic stirring bar, condenser, dropper funnel, rubber septum and nitrogen inlet quick fit on top of the condenser. The round bottom flask was placed on an ice bath followed by addition of $LiAlH_4$ (0.100 g). Separately, 2-(-2-nitroprop-1-en-1-yl)quinolin-8-ol (100 mg, 0.430 mmol) was dissolved in anhydrous tetrahydrofuran (10 ml) and transferred into the dropping funnel and the solution was added was added dropwise over a period of 30 min under a flow of nitrogen and with stirring. An additional 0.100 g of $LiAlH_4$ in tetrahydrofuran (5ml) was added dropwise via the septum. The mixture was heated at reflux for 4 h with stirring under a nitrogen atmosphere. After cooling to room temperature, the mixture was cooled further in an ice bath and the reaction was quenched by adding hydrated sodium sulfate to the flask until bubbling ceased. A further 0.200g of sodium sulfate was added and the mixture stirred for 1 h at room temperature and then filtered through celite. The solvent was evaporated using a rotary evaporator and the crude product was dissolved in dichloromethane and extracted using 5% aqueous HCl. The aqueous phase was neutralized using aqueous sodium carbonate and transferred to a separating funnel for further liquid-liquid extraction. Aqueous sodium carbonate (20%) was added until pH 11 and then the aqueous phase was further extracted with dichloromethane. The organic phase was collected, dried using anhydrous sodium sulfate, and the solvent was removed using a rotary evaporator to give a brown Solid (18.0 mg, 0.0890 mmol). Yield 20.7 %; ^1H NMR (δ, 500 MHz, $CDCl_3$) 8.04 (d, 1H, *J*=8.5Hz, H4), 7.37 (t, 1H, *J*=7.5Hz, H7), 7.27(m, 2H, H6,8), 7.14(d, 1H, *J*=7.5Hz H3), 3.70 (m, 1H, H12), 3.08-3.05 (m, 2H, H11), 1.28(d, 3H, *J*=6.5 Hz, H15); ^{13}C NMR δ: 158, 152, 138, 137, 127.1, 127, 123, 118, 111, 47.2, 46.8, 22.6; HRMS $[M+1]^+$ calc. for $[C_{12}H_{14}N_2O]^+$ 203.1179, found 203.1198; FTIR (neat) 3367, 2925, 1567, 1426, 884.0, 744.0.

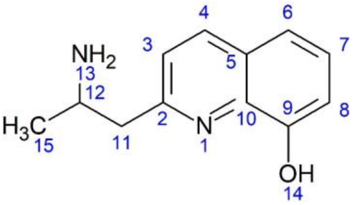

4-(2-aminopropyl)quinolin-8-ol

4-(2-aminopropyl)quinolin-8-ol was prepared using the same procedure as described above for 2-(2-aminopropyl)quinolin-8-ol but 4-(-2-nitroprop-1-en-1-yl)quinolin-8-ol (100 mg, 0.430 mmol) was used as starting material. Brown Solid (15.7 mg, 0.0780 mmol). Yield 18%; ^1H NMR (δ, 500 MHz, $CDCl_3$) 8.69 (d, 1H, *J*=4.5Hz, H2), 7.52-7.45 (m, 2H, H6,7), 7.31 (d, 1H, *J*=4.5Hz, H3), 7.17(dd, 1H, *J*=7.5, 1.5 Hz, H8), 3.4 (m, 1H, H12), 3.17-2.95 (m, 2H, H11), 1.21(d, 3H, *J*=6.5 Hz, H15); ^{13}C NMR δ: 147, 139, 128, 127, 123, 110, 48.0, 43.0, 24.0; HRMS $[M+1]^+$ calc. for $[C_{12}H_{14}N_2O]^+$ 203.1179, found 203.1191; FTIR (neat) 3330, 2932, 1625, 1454, 1371, 1316, 1090, 827.0, 598.0.

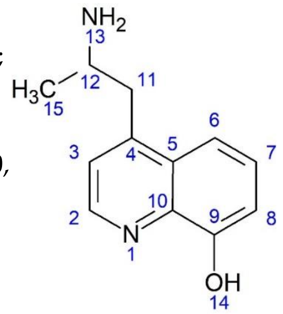

7-(2-aminopropyl)quinolin-8-ol

7-(2-aminopropyl)quinolin-8-ol was prepared using the procedure described above for 2-(2-aminopropyl)-quinolin-8-ol but 7-(-2-nitroprop-1-en-1-yl)quinolin-8-ol (100 mg, 0.430 mmol) was used as starting material. Brown solid (11.0 mg, 0.0540 mmol). Yield 14 %; ^1H NMR (δ, 500 MHz, $CDCl_3$) 8.80 (dd, 1H, *J*=1.5, 4.0Hz, H2), 8.12 (dd, 1H, *J*=1.5, 8.0Hz, H6), 7.39 (m, 1H, H3), 7.32(m, 2H, H4,7), 3.4 (m, 1H, H12), 2.99-2.84 (m, 2H, H11), 1.21(d, 3H, *J*=6.0 Hz, H15); ^{13}C NMR δ: 151, 148, 139, 136, 131, 127, 122, 121, 117, 48.0, 41.0, 24.0; HRMS $[M+1]^+$ calc. for $[C_{12}H_{14}N_2O]^+$ 203.1179, found 203.1198; FTIR (neat) 3383, 2815, 1579, 1391, 896.0, 756.0.

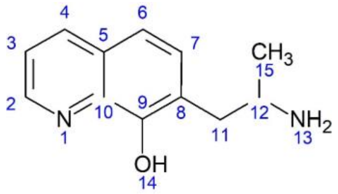

5-(2-aminopropyl)quinolin-8-ol

5-(2-aminopropyl)quinolin-8-ol was prepared using the same procedure as described above for 2-(2-aminopropyl)quinolin-8-ol but 5-(-2-nitroprop-1-en-1-yl)quinolin-8-ol (100 mg, 0.430 mmol) was used as starting material. Brown Solid (17.8 mg, 0.0880 mmol). Yield 20.6 %; ^1H NMR (δ, 500 MHz, CDCl3) 8.78 (dd, 1H, *J*=1.5, 4.5Hz, H2), 7.36 (dd, 1H, *J*1= 1Hz, *J*2= 8.5, H4), 7.46 (m, 1H, H3), 7.31(d, 1H, *J*=7.5Hz, H7), 7.13(d, 1H, *J*=7.5Hz, H8), 3.25 (m, 1H, H12), 3.01-2.86 (m, 2H, H11), 1.19(d, 3H, *J*=6.5 Hz, H15); ^{13}C NMR δ: 151, 147, 139, 133, 129, 127, 126, 121, 109, 48.1, 42.3, 23.8; HRMS $[M+1]^+$ calc. for $[C_{12}H_{14}N_2O]^+$ 203.1179, found 203.1195; FTIR (neat) 3367, 2957, 1581, 1368, 1258, 1020, 795.0.

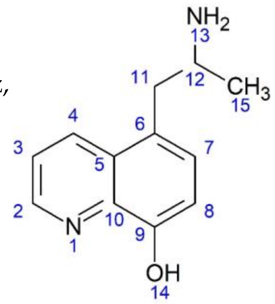

General procedure for synthesis of iron complexes of 6-10

A methanolic solution of ferric nitrate nonahydrate (1.50 mL, 0.5 mM) was added to a methanolic solution of of the quinolin-8-ol ligand (1.50 mL, 1.50 mM). The solution was stirred at ambient temperature for 15 min. The complex was precipitated by diffusion of diethyl ether into the methanolic solution in a sealed vessel.

Fe^{III}(6)$_3$: Green colour; HRMS $[M]^+$ calc. $[C_{12}H_{13}N_2O]_2Fe$ 456.1452, found 456.1342with % abund. m/z 456.1342 (6.35), 457.1250 (1.77), 458.1343 (100), 459.1374(30.05), 460.14(5.07) and $[M+1]^+$ calc. $[C_{12}H_{13}N_2O]_3Fe$ 658.2553, found 658.2344 with % abund. m/z 658.2344(6.33), 659.2500 (2.64), 660.2416(100), 661.2456(43.85), 662.2455(10.32) FTIR (neat) 3404, 2924, 1640, 1343, 1023, 829.0, 592.0; UV (10% MeOH in PBS, $\varepsilon(M^{-1}, cm^{-1})$), 597(2872), 461(3744), 366(4796), 255(69880).

Fe^{III}(7)$_3$: Green colour; HRMS $[M]^+$ calc. $[C_{12}H_{13}N_2O]_2Fe$ 456.1452, found 456.1318with % abund. m/z 456.1318 (6.35), 457.1250 (1.77), 458.1322 (100), 459.1348(30.05), 460.1400(5.07) and $[M+1]^+$ calc. $[C_{12}H_{13}N_2O]_3Fe$ 658.2553, found 658.2326 with % abund. m/z 658.2326(6.33), 659.2500 (2.64), 660.2384(100), 661.2417(43.85), 662.2455(10.32) FTIR (neat) 3410, 2927, 1635, 1358, 879.0, 753.0; UV (10% MeOH in PBS, $\varepsilon(M^{-1}, cm^{-1})$), 588(3240), 460(4992), 366(6320), 247(88000).

Fe^{III}(8)$_3$: Green crystals; HRMS $[M]^+$ calc. $[C_{12}H_{13}N_2O]_2Fe$ 456.1452, found 456.1342with % abund. m/z 456.1342 (6.35), 457.1250 (1.77), 458.1343 (100), 459.1374(30.05), 460.1400(5.07) and $[M+1]^+$ calc. [C12H13N2O]3Fe 658.2553, found 658.2344 with % abund. m/z 658.2344(6.33), 659.2500 (2.64), 660.2416(100), 661.2456(43.85), 662.2455(10.32) FTIR (neat) 3358, 2927, 1633, 1327, 830.00, 592.0; UV (10% MeOH in PBS, $\varepsilon(M^{-1}, cm^{-1})$), 600(2868), 461(3720), 366(4840), 255(69600).

Fe^{III}(9)$_3$: Green crystals; HRMS $[M]^+$ calc. $[C_{12}H_{13}N_2O]_2Fe$ 456.1452, found 456.1378 with % abund. m/z 456.1378 (6.35), 457.1250 (1.77), 458.1373 (100), 459.1404(30.05), 460.1400(5.07) and [M+1]+ calc. [C12H13N2O]3Fe 658.2553, found 658.2326 with % abund. m/z 658.2326(6.33), 659.25 (2.64), 660.2384(100), 661.2417(43.85), 662.2455(10.32) FTIR (neat) 3388, 2924, 1581, 1364, 1260, 1083, 794.0, 610.00; UV (10% MeOH in PBS, $\varepsilon(M^{-1}, cm^{-1})$), 627(2692), 473(3105), 366(4107), 255(52760).

X-Ray Crystal Structure Data

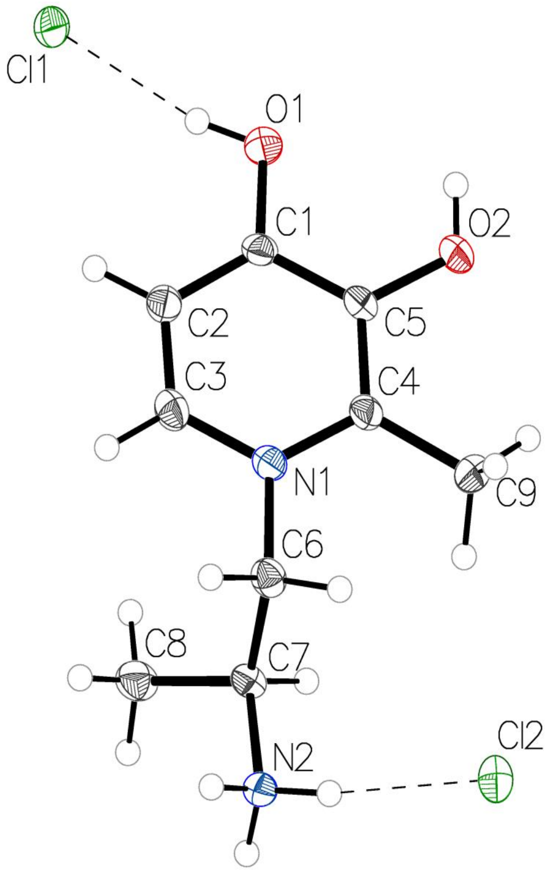

Figure S1: ORTEP diagram for **3**

Table S1: Crystallographic data for compound **3** (CCDC 2214096)

**Crystal data**	
Chemical formula	$2(Cl)\cdot C_9H_{16}N_2O_2$
M_r	255.14
Crystal system, space group	Monoclinic, $P2_1$
Temperature (K)	150
a, *b*, *c* (Å)	6.357 (9), 9.124 (12), 10.724 (17)
β (°)	96.25 (3)
V (Å³)	618.3 (15)
Z	2
Radiation type	Mo *K*α
μ (mm⁻¹)	0.51
Crystal size (mm)	× ×
**Data collection**	
Diffractometer	Bruker *APEX*-II CCD
Absorption correction	Multi-scan *SADABS2016*/2 (Bruker,2016/2) was used for absorption correction. wR2(int) was 0.1196 before and 0.0645 after correction. The Ratio of minimum to maximum transmission is 0.7787. The λ/2 correction factor is Not present.
T_{min}, T_{max}	0.581, 0.746
No. of measured, independent and observed [$I > 2\sigma(I)$] reflections	12277, 2845, 2331
R_{int}	0.072
$(\sin \theta/\lambda)_{max}$ (Å^{-1})	0.651
**Refinement**	
$R[F^2 > 2\sigma(F^2)]$, $wR(F^2)$, S	0.035, 0.068, 1.02
No. of reflections	2845
No. of parameters	147
No. of restraints	1
H-atom treatment	H atoms treated by a mixture of independent and constrained refinement
$\Delta\rho_{max}$, $\Delta\rho_{min}$ (e Å^{-3})	0.26, −0.26
Absolute structure	Flack x determined using 922 quotients [(I+)-(I-)]/[(I+)+(I-)] (Parsons, Flack and Wagner, Acta Cryst. B69 (2013) 249-259).
Absolute structure parameter	0.03 (5)

Table S2: Bond lengths and angles for **3.**

O1—C1	1.350 (4)	C3—H3	0.9500
O1—H1	0.85 (5)	C4—C5	1.392 (4)
O2—C5	1.354 (4)	C4—C9	1.487 (5)
O2—H2	0.82 (5)	C6—H6A	0.9900
N1—C3	1.354 (4)	C6—H6B	0.9900
N1—C4	1.374 (4)	C6—C7	1.539 (5)
N1—C6	1.486 (4)	C7—H7	1.0000
N2—H2A	0.9100	C7—C8	1.514 (5)
N2—H2B	0.9100	C8—H8A	0.9800
N2—H2C	0.9100	C8—H8B	0.9800
N2—C7	1.499 (4)	C8—H8C	0.9800
C1—C2	1.388 (5)	C9—H9A	0.9800
C1—C5	1.393 (4)	C9—H9B	0.9800
C2—H2D	0.9500	C9—H9C	0.9800
C2—C3	1.370 (5)		
C1—O1—H1	108 (3)	C4—C5—C1	120.7 (3)
C5—O2—H2	118 (4)	N1—C6—H6A	109.0
C3—N1—C4	121.5 (3)	N1—C6—H6B	109.0
C3—N1—C6	117.3 (3)	N1—C6—C7	112.9 (3)
C4—N1—C6	121.1 (3)	H6A—C6—H6B	107.8
H2A—N2—H2B	109.5	C7—C6—H6A	109.0
H2A—N2—H2C	109.5	C7—C6—H6B	109.0
H2B—N2—H2C	109.5	N2—C7—C6	105.8 (3)
C7—N2—H2A	109.5	N2—C7—H7	109.2
C7—N2—H2B	109.5	N2—C7—C8	109.1 (3)
C7—N2—H2C	109.5	C6—C7—H7	109.2
O1—C1—C2	124.2 (3)	C8—C7—C6	114.3 (3)
O1—C1—C5	115.9 (3)	C8—C7—H7	109.2
C2—C1—C5	119.8 (3)	C7—C8—H8A	109.5
C1—C2—H2D	120.8	C7—C8—H8B	109.5
C3—C2—C1	118.3 (3)	C7—C8—H8C	109.5
C3—C2—H2D	120.8	H8A—C8—H8B	109.5
N1—C3—C2	121.8 (3)	H8A—C8—H8C	109.5
N1—C3—H3	119.1	H8B—C8—H8C	109.5
C2—C3—H3	119.1	C4—C9—H9A	109.5
N1—C4—C5	117.8 (3)	C4—C9—H9B	109.5
N1—C4—C9	120.8 (3)	C4—C9—H9C	109.5
C5—C4—C9	121.4 (3)	H9A—C9—H9B	109.5
O2—C5—C1	122.3 (3)	H9A—C9—H9C	109.5
O2—C5—C4	117.0 (3)	H9B—C9—H9C	109.5
O1—C1—C2—C3	179.2 (3)	C3—N1—C4—C9	-178.1 (3)
O1—C1—C5—O2	0.6 (5)	C3—N1—C6—C7	85.2 (3)
O1—C1—C5—C4	-177.5 (3)	C4—N1—C3—C2	0.6 (4)
N1—C4—C5—O2	179.4 (3)	C4—N1—C6—C7	-95.4 (4)
N1—C4—C5—C1	-2.4 (4)	C5—C1—C2—C3	-0.1 (4)
N1—C6—C7—N2	175.1 (2)	C6—N1—C3—C2	180.0 (3)
N1—C6—C7—C8	-64.9 (4)	C6—N1—C4—C5	-178.2 (3)
C1—C2—C3—N1	-1.1 (4)	C6—N1—C4—C9	2.6 (4)
C2—C1—C5—O2	180.0 (3)	C9—C4—C5—O2	-1.3 (4)
C2—C1—C5—C4	1.9 (5)	C9—C4—C5—C1	176.9 (3)
C3—N1—C4—C5	1.2 (4)		

Table S3: Selected hydrogen-bond parameters for **3**.; Symmetry code(s): (i) -x+2, y-1/2, -z; (ii) -x+1, y-1/2, -z+1; (iii) x, y-1, z; (iv) x+1, y, z; (v) -x, y+1/2, -z+1.

D—H···*A*	*D*—H (Å)	H···*A* (Å)	*D*···*A* (Å)	*D*—H···*A* (°)
N2—H2A···Cl2^{i}	0.91	2.19	3.096 (4)	172.2
N2—H2B···Cl1^{ii}	0.91	2.31	3.205 (5)	170.0
N2—H2C···Cl2	0.91	2.18	3.082 (4)	172.7
C8—H8C···O2^{iii}	0.98	3.00	3.400 (6)	106.1
C9—H9A···O1^{iv}	0.98	2.62	3.502 (6)	150.1
C9—H9B···O2	0.98	2.36	2.782 (5)	105.4
O2—H2···Cl1^{v}	0.82 (5)	2.31 (5)	3.109 (4)	165 (5)
O2—H2···O1	0.82 (5)	2.42 (5)	2.707 (4)	102 (4)
O1—H1···Cl1	0.85 (5)	2.09 (5)	2.935 (4)	169 (4)

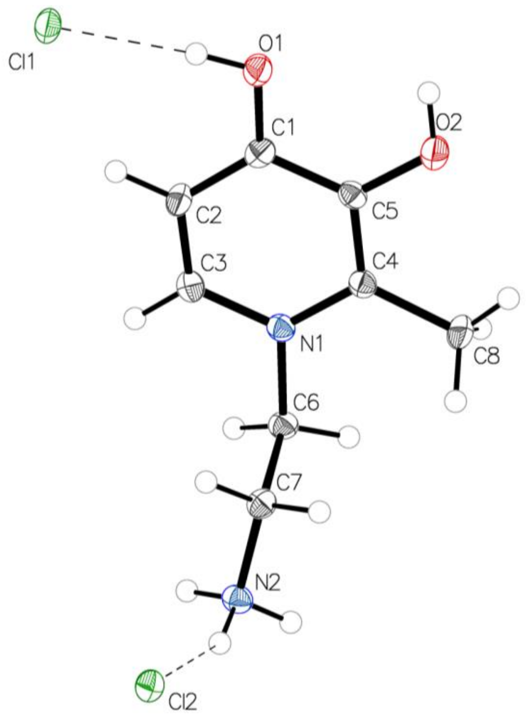

Figure S2: ORTEP diagram for **4**

Table S4: Crystallographic data for **4**. (CCDC 2214097)

**Crystal data**	
Chemical formula	$2(Cl)\cdot C_8H_{14}N_2O_2$
M_r	241.11
Crystal system, space group	Monoclinic, $P2_1/n$
Temperature (K)	150
a, *b*, *c* (Å)	4.5926 (6), 14.7686 (18), 15.2875 (19)
β (°)	92.698 (4)
V (Å^3)	1035.7 (2)
Z	4
Radiation type	Mo *K*α
μ (mm^{-1})	0.60
Crystal size (mm)	× ×
**Data collection**	
Diffractometer	Bruker *APEX*-II CCD
Absorption correction	Multi-scan *SADABS2016*/2 (Bruker,2016/2) was used for absorption correction. wR2 (int) was 0.1417 before and 0.0922 after correction. The Ratio of minimum to maximum transmission is 0.6552. The λ/2 correction factor is Not present.
T_{min}, T_{max}	0.489, 0.746
No. of measured, independent and observed [$I > 2\sigma(I)$] reflections	17507, 2378, 1888
R_{int}	0.093
(sin θ/λ)$_{max}$ (Å^{-1})	0.651
**Refinement**	
$R[F^2 > 2\sigma(F^2)]$, $wR(F^2)$, S	0.040, 0.085, 1.06
No. of reflections	2378
No. of parameters	137
H-atom treatment	H atoms treated by a mixture of independent and constrained refinement
$\Delta\rho_{max}$, $\Delta\rho_{min}$ (e Å^{-3})	0.31, −0.33

Table S5: Bond lengths and angles for **4**.

O1—C1	1.324 (3)	C2—H2D	0.9500
O1—H1	0.89 (3)	C2—C3	1.357 (3)
O2—C5	1.357 (3)	C3—H3	0.9500
O2—H2	0.87 (4)	C4—C5	1.385 (3)
N1—C3	1.365 (3)	C4—C8	1.494 (3)
N1—C4	1.361 (3)	C6—H6A	0.9900
N1—C6	1.477 (3)	C6—H6B	0.9900
N2—H2A	0.9100	C6—C7	1.523 (3)
N2—H2B	0.9100	C7—H7A	0.9900
N2—H2C	0.9100	C7—H7B	0.9900
N2—C7	1.479 (3)	C8—H8A	0.9800
C1—C2	1.387 (3)	C8—H8B	0.9800
C1—C5	1.397 (3)	C8—H8C	0.9800
C1—O1—H1	110 (2)	C5—C4—C8	121.5 (2)
C5—O2—H2	116 (3)	O2—C5—C1	121.7 (2)
C3—N1—C6	116.03 (18)	O2—C5—C4	117.55 (19)
C4—N1—C3	121.36 (19)	C4—C5—C1	120.7 (2)
C4—N1—C6	122.47 (18)	N1—C6—H6A	109.9
H2A—N2—H2B	109.5	N1—C6—H6B	109.9
H2A—N2—H2C	109.5	N1—C6—C7	108.70 (17)
H2B—N2—H2C	109.5	H6A—C6—H6B	108.3
C7—N2—H2A	109.5	C7—C6—H6A	109.9
C7—N2—H2B	109.5	C7—C6—H6B	109.9
C7—N2—H2C	109.5	N2—C7—C6	111.27 (17)
O1—C1—C2	125.0 (2)	N2—C7—H7A	109.4
O1—C1—C5	116.2 (2)	N2—C7—H7B	109.4
C2—C1—C5	118.8 (2)	C6—C7—H7A	109.4
C1—C2—H2D	120.2	C6—C7—H7B	109.4
C3—C2—C1	119.6 (2)	H7A—C7—H7B	108.0
C3—C2—H2D	120.2	C4—C8—H8A	109.5
N1—C3—H3	119.5	C4—C8—H8B	109.5
C2—C3—N1	121.0 (2)	C4—C8—H8C	109.5
C2—C3—H3	119.5	H8A—C8—H8B	109.5
N1—C4—C5	118.47 (19)	H8A—C8—H8C	109.5
N1—C4—C8	120.0 (2)	H8B—C8—H8C	109.5
O1—C1—C2—C3	-177.9 (2)	C3—N1—C4—C8	178.8 (2)
O1—C1—C5—O2	-0.1 (3)	C3—N1—C6—C7	-82.9 (2)
O1—C1—C5—C4	177.0 (2)	C4—N1—C3—C2	-0.2 (3)
N1—C4—C5—O2	179.29 (18)	C4—N1—C6—C7	92.9 (2)
N1—C4—C5—C1	2.1 (3)	C5—C1—C2—C3	1.7 (3)
N1—C6—C7—N2	165.16 (16)	C6—N1—C3—C2	175.6 (2)
C1—C2—C3—N1	-0.3 (3)	C6—N1—C4—C5	-176.24 (19)
C2—C1—C5—O2	-179.7 (2)	C6—N1—C4—C8	3.2 (3)
C2—C1—C5—C4	-2.6 (3)	C8—C4—C5—O2	-0.1 (3)
C3—N1—C4—C5	-0.6 (3)	C8—C4—C5—C1	-177.4 (2)

Table S6: Selected hydrogen-bond parameters for **4**; Symmetry code(s): (i) x+1, y, z; (ii)-x+2, -y+1, -z+1; (iii) x+1/2, -y+3/2, z+1/2; (iv) -x+3/2, y+1/2, -z+1/2; (v) -x, -y+1, -z.

D—H⋯*A*	*D*—H (Å)	H⋯*A* (Å)	*D*⋯*A* (Å)	*D*—H⋯*A* (°)
N2—H2A⋯Cl2^{i}	0.91	2.75	3.2886 (19)	119.2
N2—H2A⋯Cl2^{ii}	0.91	2.50	3.2887 (19)	145.4
N2—H2B⋯O2^{iii}	0.91	2.91	3.303 (2)	107.8
N2—H2B⋯Cl2	0.91	2.25	3.0952 (19)	153.5
N2—H2C⋯Cl1^{iv}	0.91	2.30	3.1610 (19)	158.7
C6—H6B⋯Cl2^{ii}	0.99	2.68	3.443 (2)	133.6
C8—H8B⋯O2	0.98	2.35	2.794 (3)	107.0
O1—H1⋯Cl1	0.89 (3)	2.13 (3)	3.0164 (17)	175 (3)
O2—H2⋯Cl1^{v}	0.87 (4)	2.25 (4)	3.0763 (19)	160 (4)
O2—H2⋯O1	0.87 (4)	2.36 (4)	2.694 (2)	103 (3)

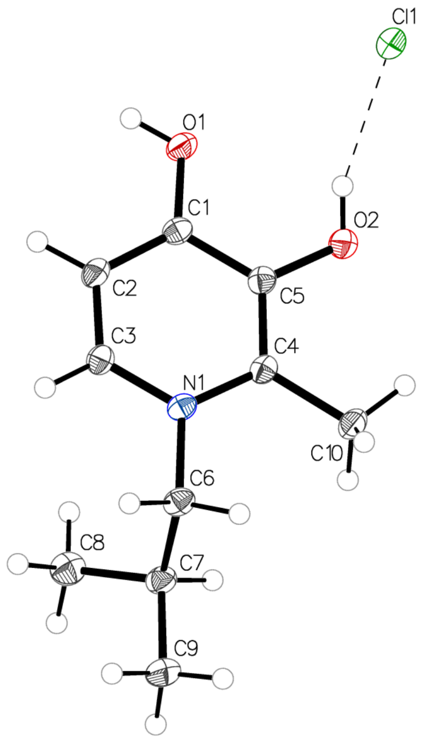

Figure S3: ORTEP diagram for **5**

Table S7: Crystallographic data for **5** (CCDC 2214098).

**Crystal data**	
Chemical formula	$Cl \cdot C_{10}H_{16}NO_2$
M_r	217.69
Crystal system, space group	Triclinic, *P*1
Temperature (K)	150
a, *b*, *c* (Å)	7.656 (3), 8.364 (3), 9.385 (6)
α, β, γ (°)	85.387 (13), 77.367 (18), 69.698 (9)
V (Å^3)	550.0 (5)
Z	2
Radiation type	Mo *K*α
μ (mm^{-1})	0.32
Crystal size (mm)	× ×
**Data collection**	
Diffractometer	Bruker *APEX*-II CCD
Absorption correction	Multi-scan *SADABS2016*/2 (Bruker,2016/2) was used for absorption correction. wR2(int) was 0.1345 before and 0.0964 after correction. The Ratio of minimum to maximum transmission is 0.7373. The λ/2 correction factor is Not present.
T_{min}, T_{max}	0.550, 0.746
No. of measured, independent and observed [$I > 2\sigma(I)$] reflections	21075, 2512, 2351
R_{int}	0.064
$(\sin \theta/\lambda)_{max}$ (Å^{-1})	0.650
**Refinement**	
$R[F^2 > 2\sigma(F^2)]$, $wR(F^2)$, S	0.033, 0.093, 1.09
No. of reflections	2512
No. of parameters	138
H-atom treatment	H atoms treated by a mixture of independent and constrained refinement
$\Delta\rho_{max}$, $\Delta\rho_{min}$ (e Å^{-3})	0.44, −0.40

Table S8: Bond lengths and angles for 5.

O1—C1	1.3301 (15)	C2—C3	1.3720 (17)
O2—C5	1.3563 (15)	C4—C5	1.3894 (17)
N1—C3	1.3528 (16)	C4—C10	1.4968 (17)
N1—C4	1.3712 (16)	C6—C7	1.5327 (17)
N1—C6	1.4909 (15)	C7—C8	1.524 (2)
C1—C2	1.3980 (17)	C7—C9	1.5340 (18)
C1—C5	1.4093 (17)		
C3—N1—C4	121.19 (10)	N1—C4—C10	120.17 (11)
C3—N1—C6	117.45 (10)	C5—C4—C10	121.10 (11)
C4—N1—C6	121.36 (10)	O2—C5—C1	120.92 (11)
O1—C1—C2	124.68 (11)	O2—C5—C4	118.44 (11)
O1—C1—C5	116.80 (11)	C4—C5—C1	120.61 (11)
C2—C1—C5	118.51 (11)	N1—C6—C7	113.61 (10)
C3—C2—C1	119.19 (11)	C6—C7—C9	107.30 (11)
N1—C3—C2	121.73 (11)	C8—C7—C6	112.26 (10)
N1—C4—C5	118.73 (11)	C8—C7—C9	109.99 (11)
O1—C1—C2—C3	-179.00 (11)	C3—N1—C4—C10	-178.88 (10)
O1—C1—C5—O2	-1.23 (16)	C3—N1—C6—C7	89.52 (13)
O1—C1—C5—C4	-179.21 (10)	C4—N1—C3—C2	0.56 (18)
N1—C4—C5—O2	-179.99 (10)	C4—N1—C6—C7	-89.81 (13)
N1—C4—C5—C1	-1.95 (16)	C5—C1—C2—C3	0.89 (17)
N1—C6—C7—C8	-60.57 (14)	C6—N1—C3—C2	-178.77 (10)
N1—C6—C7—C9	178.48 (10)	C6—N1—C4—C5	-179.45 (10)
C1—C2—C3—N1	-1.64 (18)	C6—N1—C4—C10	0.42 (16)
C2—C1—C5—O2	178.88 (11)	C10—C4—C5—O2	0.14 (16)
C2—C1—C5—C4	0.89 (17)	C10—C4—C5—C1	178.17 (10)
C3—N1—C4—C5	1.25 (16)		

Table S9: Selected hydrogen-bond parameters for **5**; Symmetry code(s): (i) -x+1, -y+1, -z+1; (ii) -x+2, -y+1, -z+1.

D—H···*A*	*D*—H (Å)	H···*A* (Å)	*D*···*A* (Å)	*D*—H···*A* (°)
C8—H8C···O1^{i}	0.98	2.61	3.437 (2)	142.6
C10—H10B···O2	0.98	2.35	2.8099 (19)	108.1
O2—H2···Cl1	0.83 (2)	2.26 (2)	3.0511 (16)	158.5 (18)
O2—H2···O1	0.83 (2)	2.39 (2)	2.7061 (17)	103.3 (15)
O1—H1···Cl1^{ii}	0.84 (2)	2.13 (2)	2.9701 (14)	179 (2)

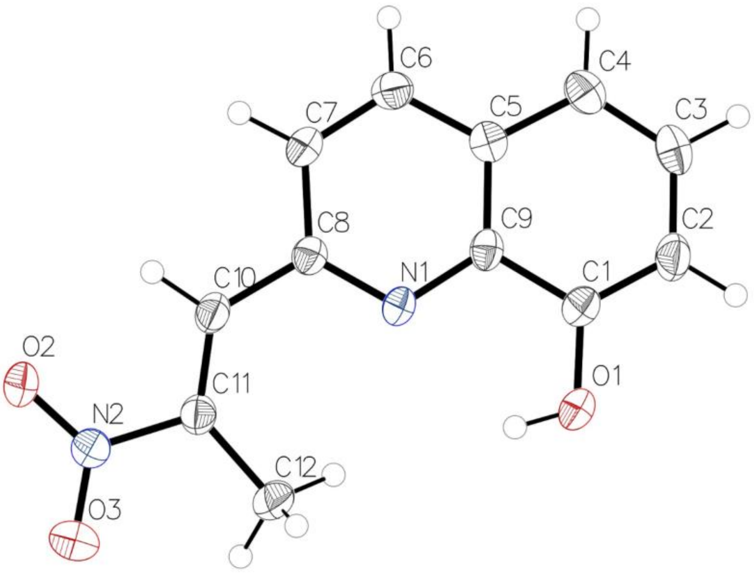

Figure S4: ORTEP diagram for 2-(-2-nitroprop-1-en-1-yl)quinoline-8-ol

Table S10. Crystallographic data for 2-(-2-nitroprop-1-en-1-yl)quinoline-8-ol (CCDC 2204668)

**Crystal Data**	
Chemical formula	$C_{12}H_{10}N_2O_3$
M_r	230.22
Crystal system, space group	Monoclinic, $P2_1/n$
Temperature (K)	150
a, *b*, *c* (Å)	13.0598 (12), 6.3565 (6), 13.7985 (13)
β (°)	110.237 (4)
V (Å^3)	1074.77 (18)
Z	4
Radiation type	Mo $K\alpha$
μ (mm^{-1})	0.11
Crystal size (mm)	0.18 × 0.08 × 0.03
**Data collection**	
Diffractometer	Bruker D8Quest
Absorption correction	Multi-scan *SADABS2016*/2 (Bruker,2016/2) was used for absorption correction. wR2 (int) was 0.1752 before and 0.0900 after correction. The Ratio of minimum to maximum transmission is 0.9115. The λ/2 correction factor is Not present.
T_{min}, T_{max}	0.680, 0.746
No. of measured, independent and observed [$I > 2\sigma(I)$] reflections	26876, 2473, 1731
R_{int}	0.098
$(\sin \theta/\lambda)_{max}$ (Å^{-1})	0.651
**Refinement**	
$R[F^2 > 2\sigma(F^2)]$, $wR(F^2)$, S	0.058, 0.131, 1.06
No. of reflections	2473
No. of parameters	156
H-atom treatment	H-atom parameters constrained
$\Delta\rho_{max}$, $\Delta\rho_{min}$ (e Å^{-3})	0.29, −0.27

Table S11: Bond length and angles for 2-(-2-nitroprop-1-en-1-yl)quinoline-8-ol

C1—O1	1.355 (3)	C4—C5	1.419 (3)
C1—C2	1.364 (3)	C5—C6	1.408 (3)
C1—C9	1.421 (3)	C5—C9	1.411 (3)
N1—C8	1.325 (3)	C6—H6	0.9500
N1—C9	1.358 (3)	C6—C7	1.363 (3)
O1—H1	0.8400	C7—H7	0.9500
C2—H2	0.9500	C7—C8	1.417 (3)
C2—C3	1.403 (3)	C8—C10	1.466 (3)
N2—O2	1.222 (2)	C10—H10	0.9500
N2—O3	1.224 (2)	C10—C11	1.328 (3)
N2—C11	1.473 (3)	C11—C12	1.486 (3)
C3—H3	0.9500	C12—H12A	0.9800
C3—C4	1.368 (3)	C12—H12B	0.9800
C4—H4	0.9500	C12—H12C	0.9800
O1—C1—C2	121.5 (2)	C7—C6—H6	120.3
O1—C1—C9	118.1 (2)	C6—C7—H7	119.9
C2—C1—C9	120.4 (2)	C6—C7—C8	120.1 (2)
C8—N1—C9	117.67 (18)	C8—C7—H7	119.9
C1—O1—H1	109.5	N1—C8—C7	122.1 (2)
C1—C2—H2	120.2	N1—C8—C10	119.90 (19)
C1—C2—C3	119.6 (2)	C7—C8—C10	117.99 (19)
C3—C2—H2	120.2	N1—C9—C1	116.24 (19)
O2—N2—O3	121.9 (2)	N1—C9—C5	124.10 (19)
O2—N2—C11	119.90 (19)	C5—C9—C1	119.7 (2)
O3—N2—C11	118.17 (19)	C8—C10—H10	116.0
C2—C3—H3	119.0	C11—C10—C8	128.0 (2)
C4—C3—C2	121.9 (2)	C11—C10—H10	116.0
C4—C3—H3	119.0	N2—C11—C12	114.56 (19)
C3—C4—H4	120.2	C10—C11—N2	115.06 (19)
C3—C4—C5	119.5 (2)	C10—C11—C12	130.4 (2)
C5—C4—H4	120.2	C11—C12—H12A	109.5
C6—C5—C4	124.5 (2)	C11—C12—H12B	109.5
C6—C5—C9	116.6 (2)	C11—C12—H12C	109.5
C9—C5—C4	118.9 (2)	H12A—C12—H12B	109.5
C5—C6—H6	120.3	H12A—C12—H12C	109.5
C7—C6—C5	119.3 (2)	H12B—C12—H12C	109.5

Table S12: Selected hydrogen-bond parameters for 2-(-2-nitroprop-1-en-1-yl)quinoline-8-ol; Symmetry code(s): (i) -x+2, -y+1, -z+1; (ii) -x+2, -y, -z+1; (iii) x-1/2, -y+3/2, z-1/2; (iv) -x+1, -y+2, -z+1.

D—H···*A*	*D*—H (Å)	H···*A* (Å)	*D*···*A* (Å)	*D*—H···*A* (°)
O1—H1···N1	0.84	2.18	2.660 (2)	116.4
O1—H1···O1^{i}	0.84	2.73	3.127 (4)	110.5
C2—H2···O1^{ii}	0.95	2.52	3.354 (3)	146.1
C6—H6···O3^{iii}	0.95	2.49	3.305 (3)	144.1
C7—H7···O2^{iv}	0.95	2.47	3.349 (3)	153.4
C10—H10···O2	0.95	2.24	2.652 (3)	105.5
C12—H12B···N1	0.98	2.40	2.986 (3)	117.9

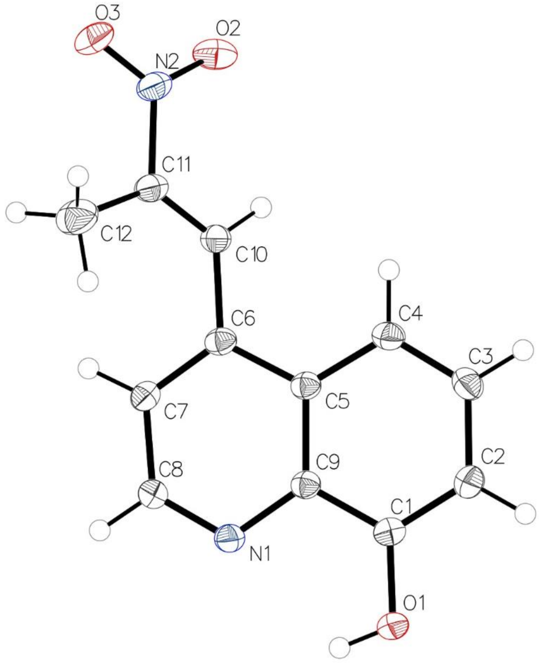

Figure S5: ORTEP diagram for 4-(-2-nitroprop-1-en-1-yl)quinoline-8-ol

Table S13: Crystallographic data for 4-(-2-nitroprop-1-en-1-yl)quinoline-8-ol (CCDC 2204672)

**Crystal data**	
Chemical formula	$C_{12}H_{10}N_2O_3$
M_r	230.22
Crystal system, space group	Triclinic, *P*1
Temperature (K)	150
a, *b*, *c* (Å)	6.6710 (3), 8.0412 (4), 10.5537 (6)
α, β, γ (°)	100.492 (2), 90.759 (2), 108.475 (2)
V ($Å^3$)	526.45 (5)
Z	2
Radiation type	Mo *K*α
μ (mm^{-1})	0.11
Crystal size (mm)	0.41 × 0.09 × 0.03
**Data collection**	
Diffractometer	Bruker D8Quest
Absorption correction	Multi-scan *SADABS2016*/2 (Bruker,2016/2) was used for absorption correction. wR2 was 0.1607 before and 0.0769 after correction. The Ratio of minimum to maximum transmission is 0.7562. The λ/2 correction factor is Not present.
T_{min}, T_{max}	0.564, 0.746
No. of measured, independent and observed [$I > 2\sigma(I)$] reflections	42186, 2449, 2106
R_{int}	0.059
$(\sin \theta/\lambda)_{max}$ ($Å^{-1}$)	0.654
**Refinement**	
$R[F^2 > 2\sigma(F^2)]$, $wR(F^2)$, S	0.035, 0.106, 1.08
No. of reflections	2449
No. of parameters	156
H-atom treatment	H-atom parameters constrained
$\Delta\rho_{max}$, $\Delta\rho_{min}$ (e $Å^{-3}$)	0.27, −0.27

Table S14: Bond lengths and angles for 4-(-2-nitroprop-1-en-1-yl)quinoline-8-ol

C1—O1	1.3563 (13)	C3—C4	1.3699 (17)
C1—C2	1.3746 (16)	C4—C5	1.4174 (16)
C1—C9	1.4255 (15)	C5—C6	1.4276 (15)
N1—C8	1.3202 (15)	C5—C9	1.4249 (14)
N1—C9	1.3695 (14)	C6—C7	1.3729 (16)
C2—C3	1.4104 (16)	C6—C10	1.4790 (14)
N2—O2	1.2305 (14)	C7—C8	1.4119 (15)
N2—O3	1.2250 (14)	C10—C11	1.3279 (16)
N2—C11	1.4854 (14)	C11—C12	1.4836 (17)
O1—C1—C2	118.88 (10)	C5—C6—C10	120.03 (10)
O1—C1—C9	120.76 (10)	C7—C6—C5	118.64 (10)
C2—C1—C9	120.35 (10)	C7—C6—C10	121.28 (10)
C8—N1—C9	117.17 (9)	C6—C7—C8	119.67 (10)
C1—C2—C3	119.95 (11)	N1—C8—C7	124.00 (10)
O2—N2—C11	119.52 (10)	N1—C9—C1	117.70 (10)
O3—N2—O2	123.17 (10)	N1—C9—C5	123.38 (10)
O3—N2—C11	117.30 (11)	C5—C9—C1	118.91 (10)
C4—C3—C2	121.54 (11)	C11—C10—C6	123.42 (10)
C3—C4—C5	119.67 (10)	C10—C11—N2	116.01 (10)
C4—C5—C6	123.34 (10)	C10—C11—C12	129.95 (11)
C4—C5—C9	119.56 (10)	C12—C11—N2	114.03 (10)
C9—C5—C6	117.10 (10)		

Table S15: Selected hydrogen-bond parameters for 4-(-2-nitroprop-1-en-1-yl)quinoline-8-ol; Symmetry code(s): (i) -x+2, -y+1, -z.

D—H···*A*	*D*—H (Å)	H···*A* (Å)	*D*···*A* (Å)	*D*—H···*A* (°)
O1—H1···N1	0.84	2.30	2.7562 (13)	114.4
O1—H1···N1^{i}	0.84	2.19	2.8511 (13)	135.4
C8—H8···O1^{i}	0.95	2.46	3.0005 (14)	116.2
C10—H10···O2	0.95	2.30	2.6768 (14)	102.8

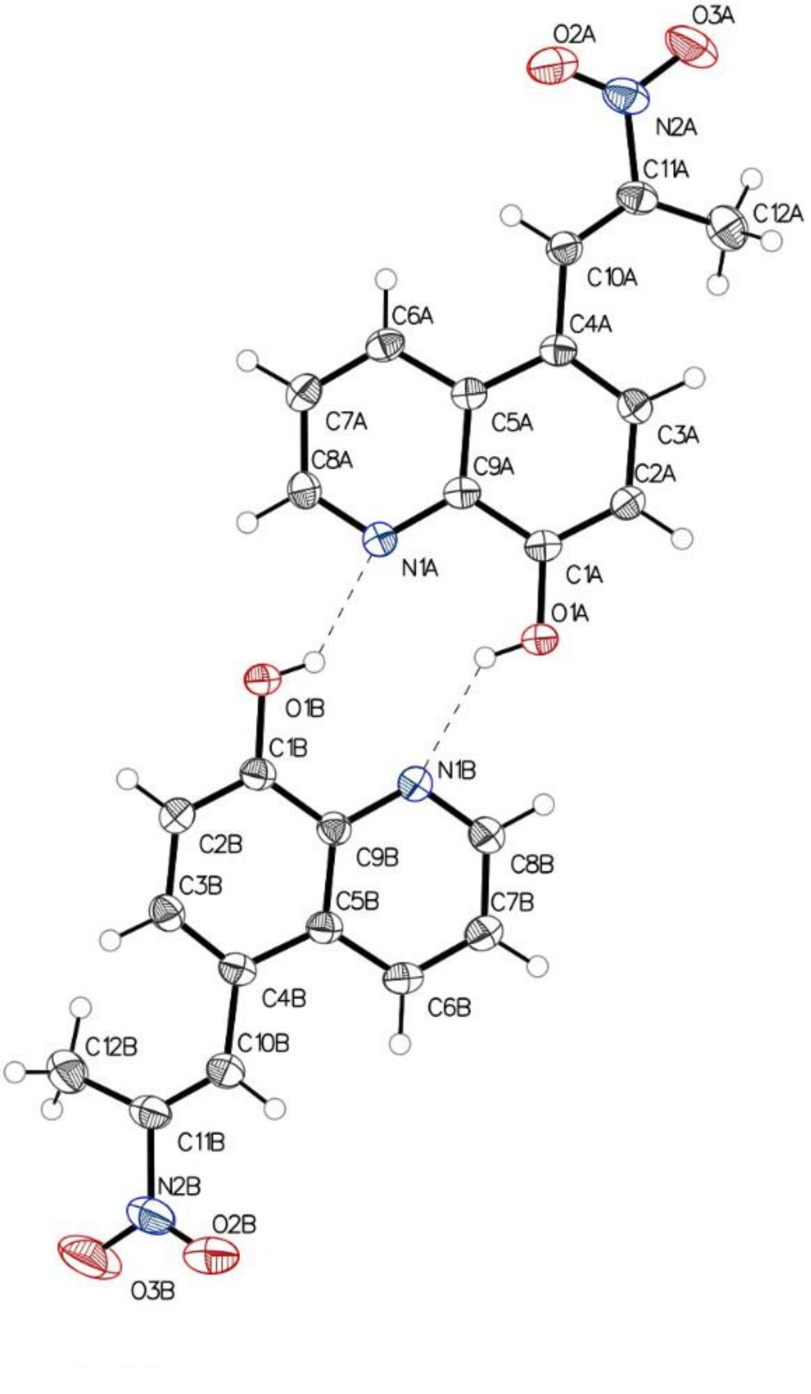

Figure S6: ORTEP diagram for 5-(-2-nitroprop-1-en-1-yl)quinoline-8-ol

Table S16: Crystallographic data for 5-(-2-nitroprop-1-en-1-yl)quinoline-8-ol (CCDC 2204673)

**Crystal data**	
Chemical formula	$2(C_{12}H_{10}N_2O_3)$
M_r	460.44
Crystal system, space group	Triclinic, *P*1bar
Temperature (K)	150
a, *b*, *c* (Å)	7.0189 (13), 10.9644 (19), 14.234 (3)
α, β, γ (°)	80.648 (6), 88.802 (6), 75.050 (6)
V (Å^3)	1044.0 (3)
Z	2
Radiation type	Mo *K*α
μ (mm^{-1})	0.11
Crystal size (mm)	0.15 × 0.11 × 0.09
**Data collection**	
Diffractometer	Bruker D8Quest
Absorption correction	Multi-scan *SADABS2016*/2 (Bruker,2016/2) was used for absorption correction. wR2(int) was 0.1385 before and 0.0899 after correction. The Ratio of minimum to maximum transmission is 0.8765. The λ/2 correction factor is Not present.
T_{min}, T_{max}	0.654, 0.746
No. of measured, independent and observed [$I > 2\sigma(I)$] reflections	45104, 4817, 2810
R_{int}	0.120
$(\sin \theta/\lambda)_{max}$ (Å^{-1})	0.652
**Refinement**	
$R[F^2 > 2\sigma(F^2)]$, $wR(F^2)$, S	0.057, 0.154, 1.02
No. of reflections	4817
No. of parameters	311
H-atom treatment	H-atom parameters constrained
$\Delta\rho_{max}$, $\Delta\rho_{min}$ (e Å^{-3})	0.31, −0.32

Table S17: Bond lengths and angles for 5-(-2-nitroprop-1-en-1-yl)quinoline-8-ol

C1A—O1A	1.348 (3)	C1B—O1B	1.359 (3)
C1A—C2A	1.370 (3)	C1B—C2B	1.367 (3)
C1A—C9A	1.420 (3)	C1B—C9B	1.415 (3)
N1A—C8A	1.317 (3)	N1B—C8B	1.318 (3)
N1A—C9A	1.369 (3)	N1B—C9B	1.372 (3)
C2A—C3A	1.397 (3)	C2B—C3B	1.405 (3)
N2A—O2A	1.221 (3)	N2B—O2B	1.227 (3)
N2A—O3A	1.224 (3)	N2B—O3B	1.223 (3)
N2A—C11A	1.475 (3)	N2B—C11B	1.485 (3)
C3A—C4A	1.384 (3)	C3B—C4B	1.374 (4)
C4A—C5A	1.431 (3)	C4B—C5B	1.436 (3)
C4A—C10A	1.464 (3)	C4B—C10B	1.472 (3)
C5A—C6A	1.417 (3)	C5B—C6B	1.413 (4)
C5A—C9A	1.428 (3)	C5B—C9B	1.424 (3)
C6A—C7A	1.364 (3)	C6B—C7B	1.364 (4)
C7A—C8A	1.407 (3)	C7B—C8B	1.409 (3)
C10A—C11A	1.335 (3)	C10B—C11B	1.329 (4)
C11A—C12A	1.485 (4)	C11B—C12B	1.490 (4)
O1A—C1A—C2A	118.3 (2)	O1B—C1B—C2B	118.2 (2)
O1A—C1A—C9A	121.7 (2)	O1B—C1B—C9B	121.7 (2)
C2A—C1A—C9A	120.1 (2)	C2B—C1B—C9B	120.1 (2)
C8A—N1A—C9A	117.61 (19)	C8B—N1B—C9B	117.8 (2)
C1A—C2A—C3A	120.6 (2)	C1B—C2B—C3B	120.3 (2)
O2A—N2A—O3A	123.1 (2)	O2B—N2B—C11B	119.5 (2)
O2A—N2A—C11A	118.8 (2)	O3B—N2B—O2B	122.8 (2)
O3A—N2A—C11A	118.1 (2)	O3B—N2B—C11B	117.8 (3)
C4A—C3A—C2A	122.2 (2)	C4B—C3B—C2B	122.0 (2)
C3A—C4A—C5A	118.2 (2)	C3B—C4B—C5B	118.6 (2)
C3A—C4A—C10A	122.4 (2)	C3B—C4B—C10B	121.3 (2)
C5A—C4A—C10A	119.2 (2)	C5B—C4B—C10B	120.1 (2)
C6A—C5A—C4A	124.2 (2)	C6B—C5B—C4B	123.8 (2)
C6A—C5A—C9A	116.2 (2)	C6B—C5B—C9B	117.0 (2)
C9A—C5A—C4A	119.6 (2)	C9B—C5B—C4B	119.2 (2)
C7A—C6A—C5A	120.2 (2)	C7B—C6B—C5B	119.9 (2)
C6A—C7A—C8A	119.1 (2)	C6B—C7B—C8B	119.0 (2)
N1A—C8A—C7A	123.8 (2)	N1B—C8B—C7B	123.8 (2)
C1A—C9A—C5A	119.3 (2)	C1B—C9B—C5B	119.7 (2)
N1A—C9A—C1A	117.57 (19)	N1B—C9B—C1B	117.9 (2)
N1A—C9A—C5A	123.1 (2)	N1B—C9B—C5B	122.5 (2)
C11A—C10A—C4A	126.8 (2)	C11B—C10B—C4B	124.5 (2)
N2A—C11A—C12A	114.6 (2)	N2B—C11B—C12B	114.2 (2)
C10A—C11A—N2A	115.5 (2)	C10B—C11B—N2B	115.7 (2)
C10A—C11A—C12A	129.9 (2)	C10B—C11B—C12B	130.0 (2)

Table S18: Selected hydrogen-bond parameters for 5-(-2-nitroprop-1-en-1-yl)quinoline-8-ol; Symmetry code(s): (i) x, y+1, z-1; (ii) -x+2, -y+1, -z+1.

D—H···*A*	*D*—H (Å)	H···*A* (Å)	*D*···*A* (Å)	*D*—H···*A* (°)
O1A—H1A···N1A	0.84	2.31	2.762 (3)	114.1
O1A—H1A···N1B	0.84	2.09	2.772 (3)	138.0
C8A—H8A···O1B	0.95	2.60	3.020 (3)	107.1
C10A—H10A···O2A	0.95	2.24	2.641 (3)	104.6
C12A—H12A···O3A	0.98	2.30	2.685 (4)	102.5
O1B—H1B···N1A	0.84	2.09	2.776 (2)	139.0
O1B—H1B···N1B	0.84	2.33	2.771 (3)	113.7
C6B—H6B···O2A^{i}	0.95	2.30	3.243 (3)	170.3
C8B—H8B···O1A	0.95	2.56	3.014 (3)	109.4
C10B—H10B···O2A^{i}	0.95	2.54	3.309 (4)	138.1
C10B—H10B···O2B	0.95	2.30	2.675 (3)	102.8
C12B—H12E···O3A^{ii}	0.98	2.65	3.361 (3)	129.6

UV-visible spectroscopy of Fe complexes

Iron nitrate (3mM, 25µl) in 10% methanol in PBS was added to 150µl of sample solutions (**1-9**) with different concentrations (0.25 mM to 3mM) in 10% methanol in PBS using 96 well microplates. Then 125 µL of 10% methanol in PBS was added to each well. Spectral changes were measured as a correlation of ligand-metal binding between 200-800 nm at 25 °C using Agilent Technologies Cary 60 spectrometer (Santa Clara, CA, USA) with 0.1 cm path length quartz cuvette.

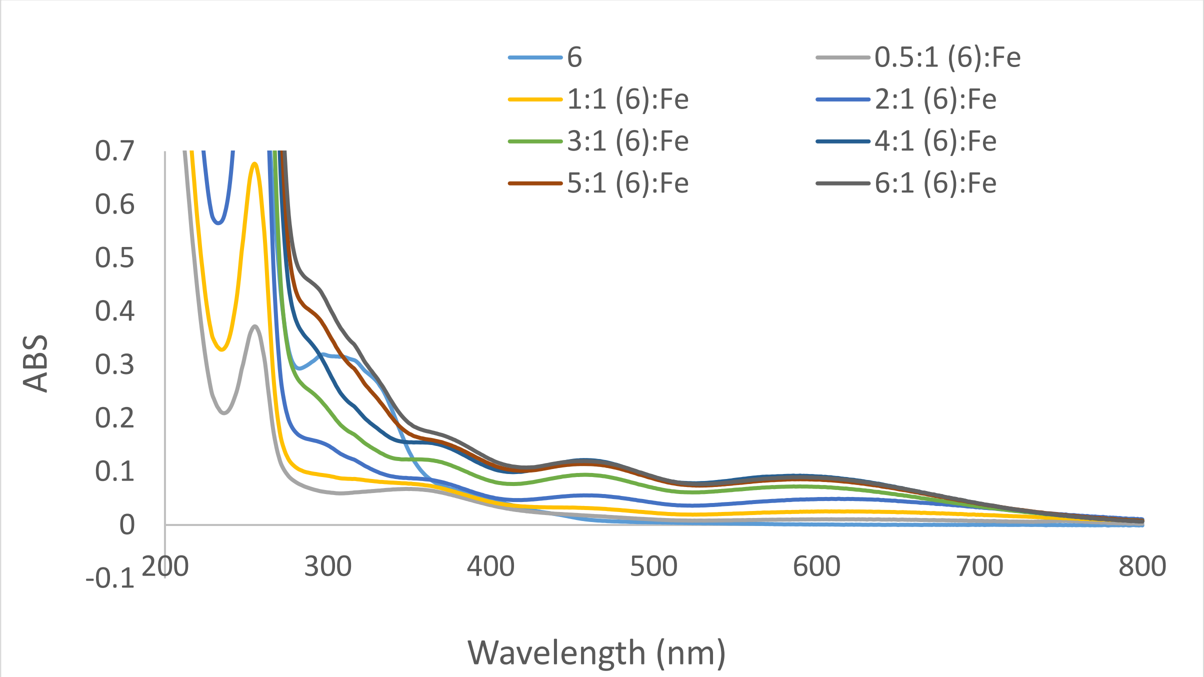

Figure S7: UV-visible spectrum of **6**:Fe complex in 10% MeOH in PBS with **6**:Fe ratio from 0.5:1 to 6:1

Isothermal Calorimetry Data

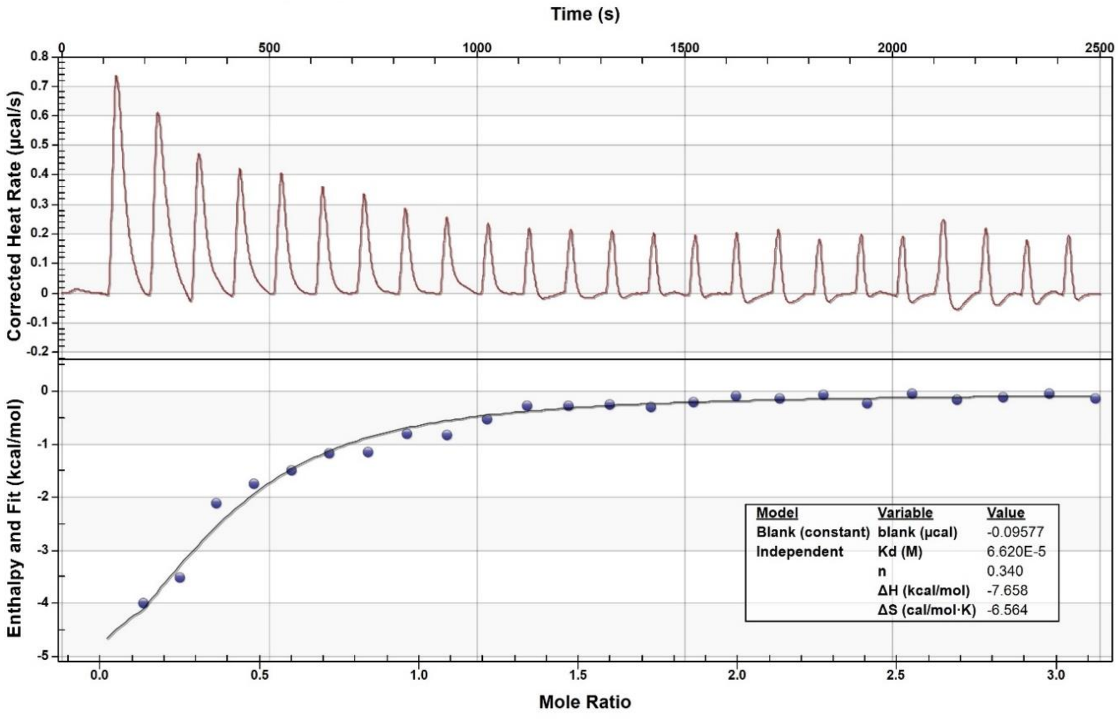

Figure S8: ITC data for iron(III) nitrate titrated into **1** (3 molar equiv.)

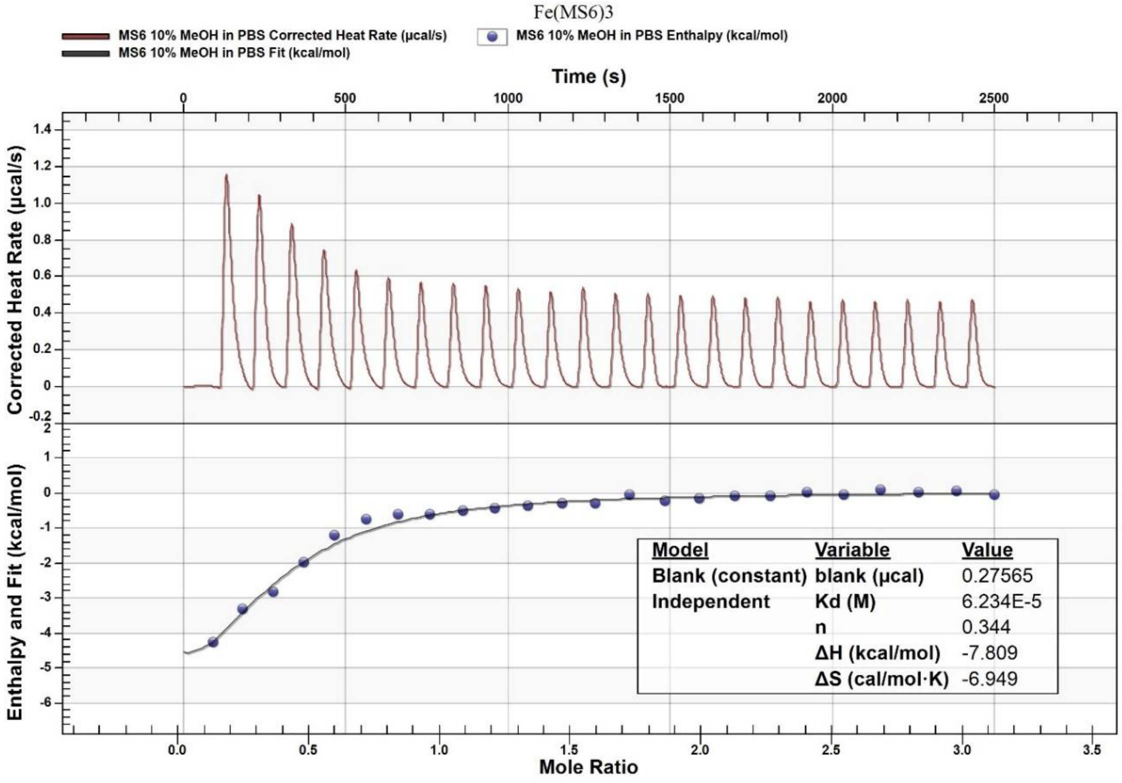

Figure S9: ITC data for iron(III) nitrate titrated into **2** (3 molar equiv.)

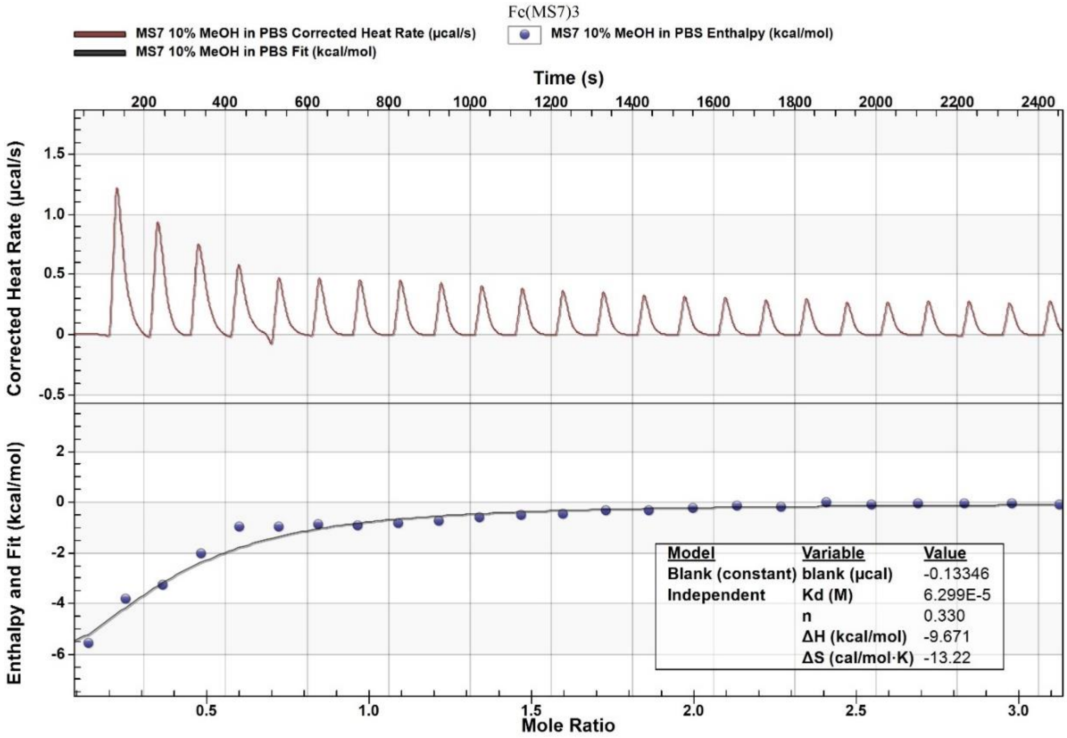

Figure S10: ITC data for iron(III) nitrate titrated into **3** (3 molar equiv.)

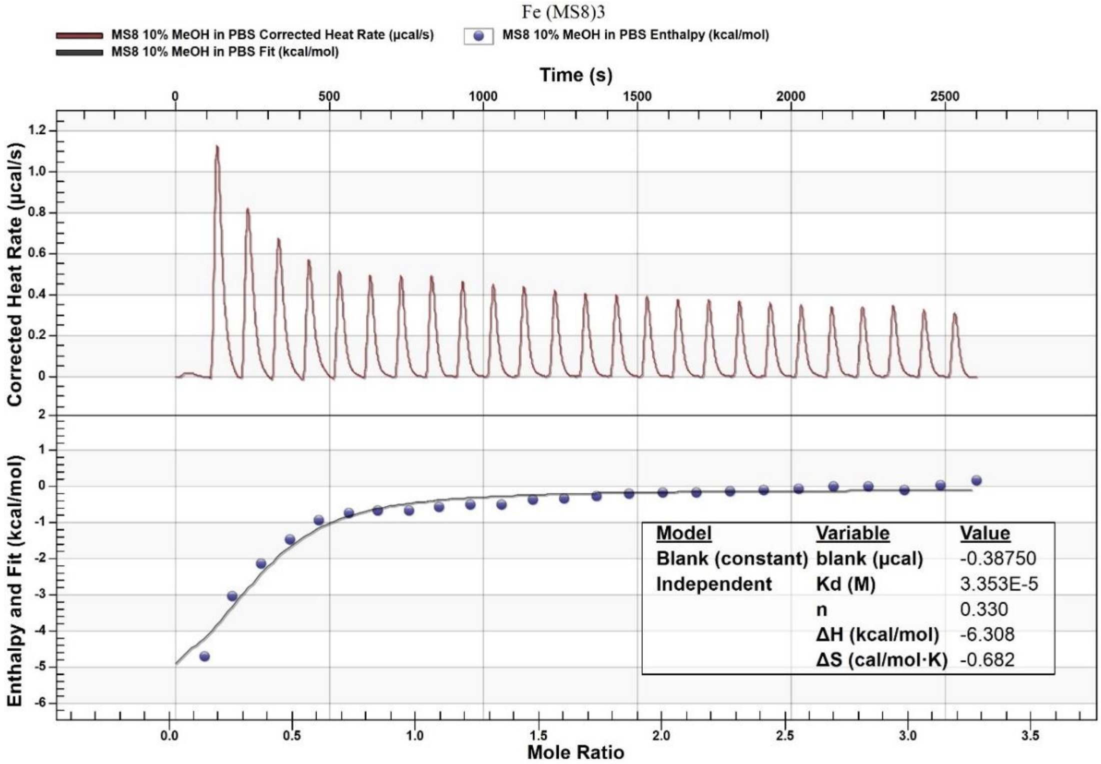

Figure S11: ITC data for iron(III) nitrate titrated into **4** (3 molar equiv.)

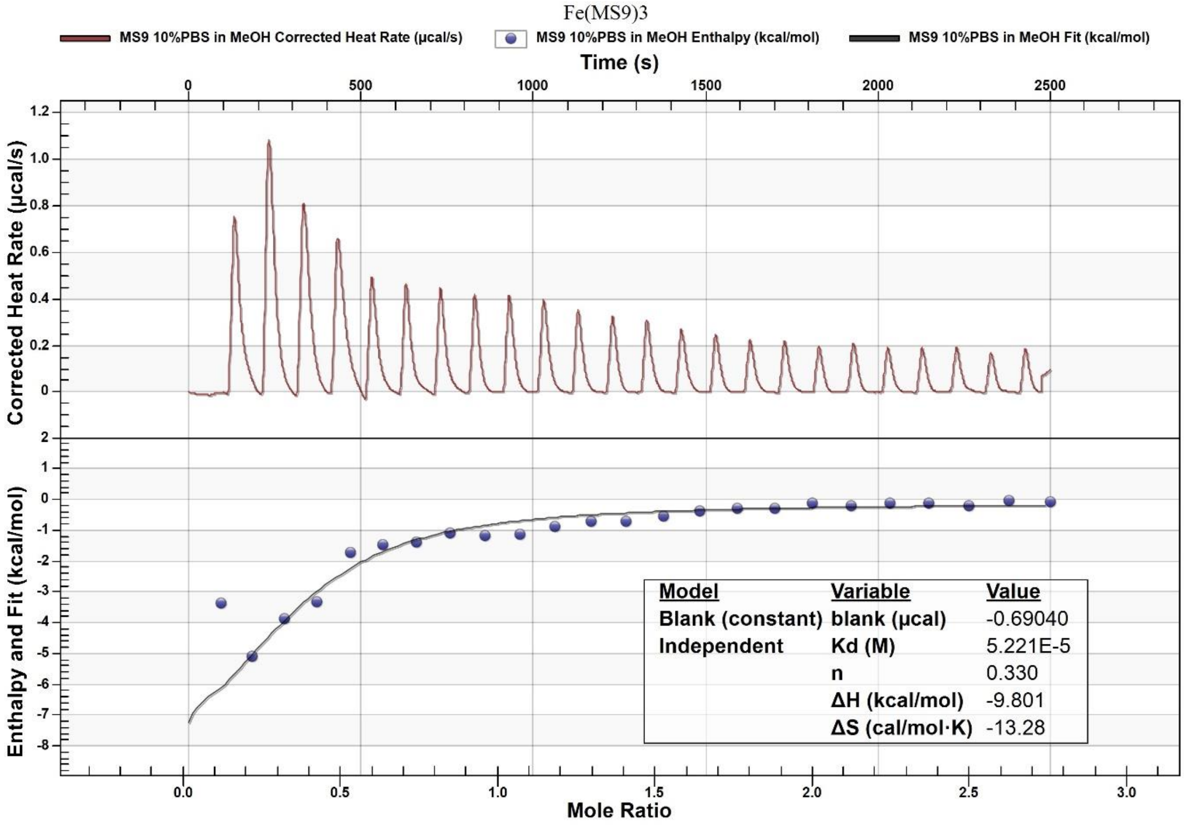

Figure S12: ITC data for iron(III) nitrate titrated into **5** (3 molar equiv.)

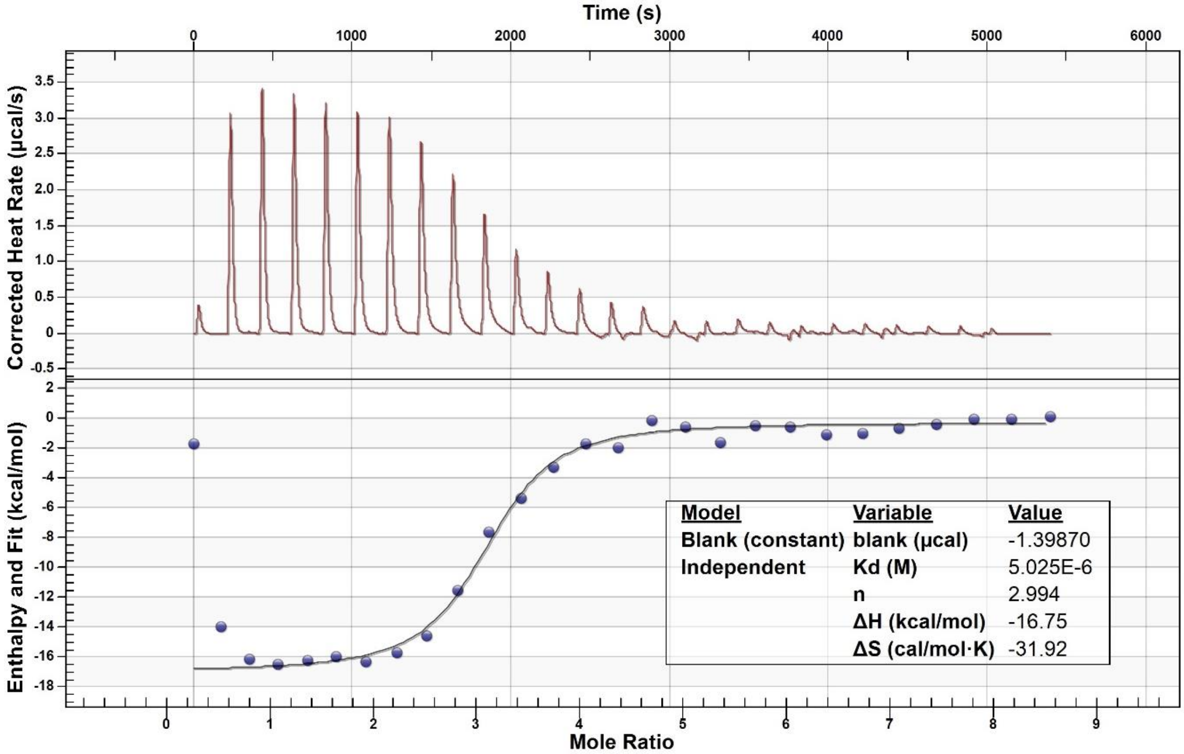

Figure S13: ITC data for **6** (3 molar equiv.) titrated into iron(III) nitrate.

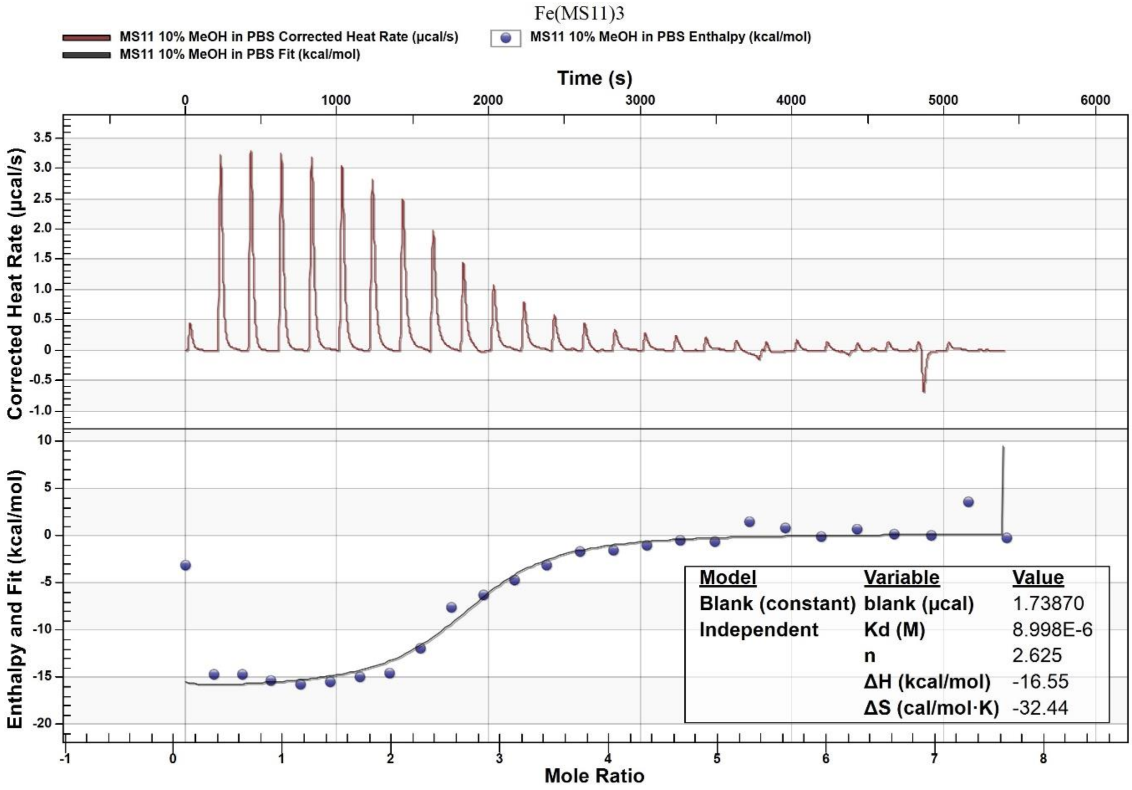

Figure S14: ITC data for **7** (3 molar equiv.) titrated into iron(III) nitrate.

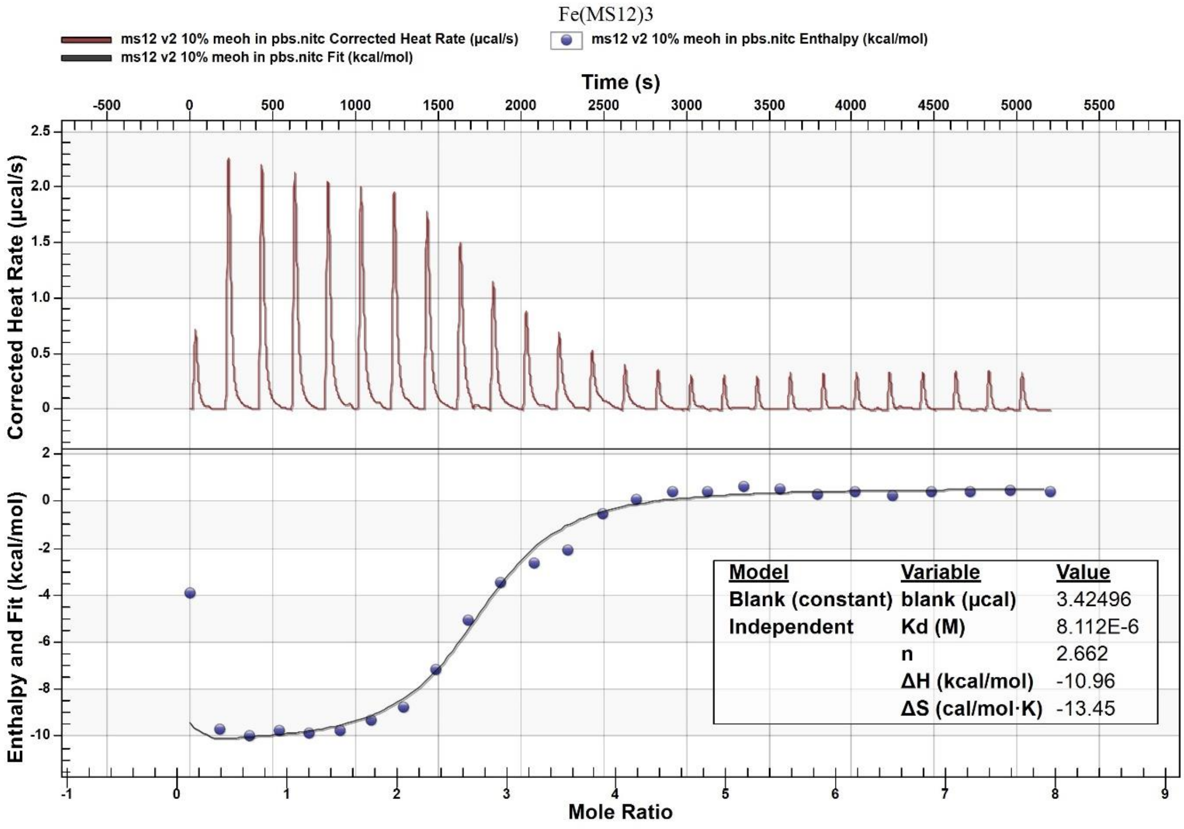

Figure S15: ITC data for **8** (3 molar equiv.) titrated into iron(III) nitrate.

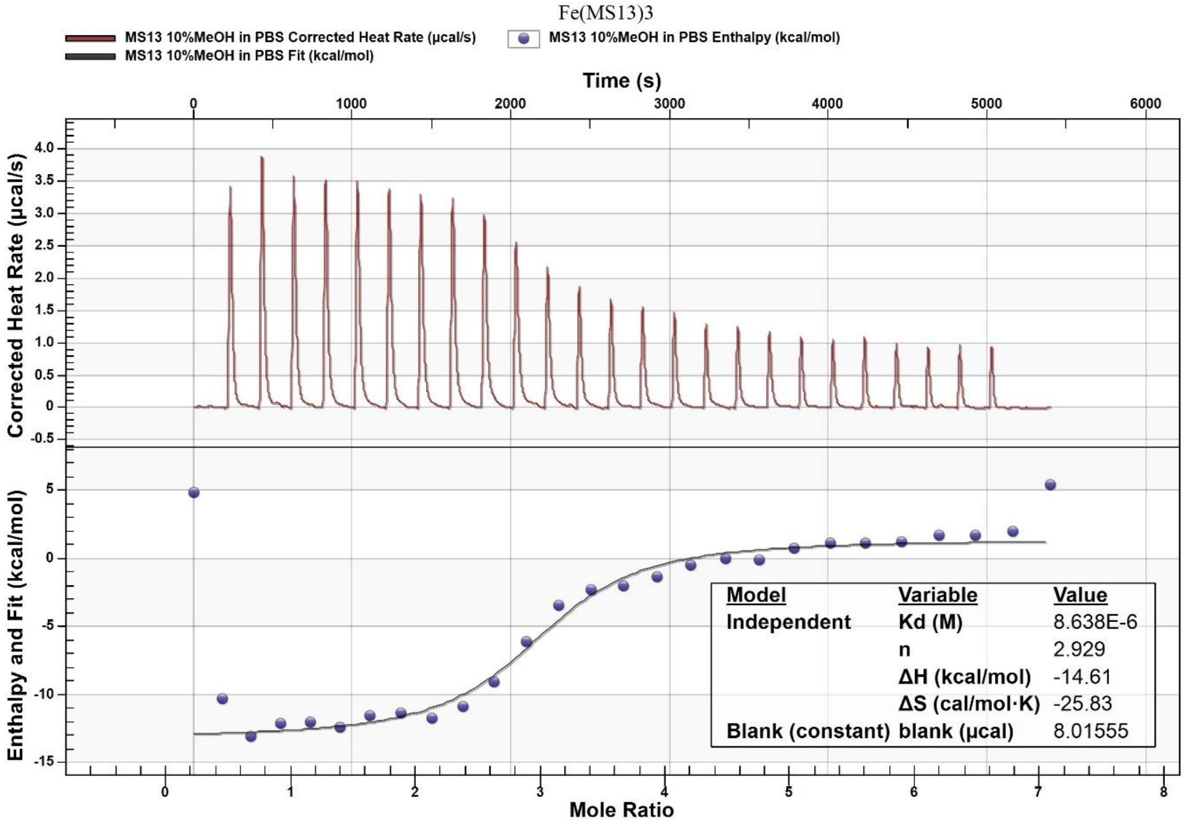

Figure S16: ITC data for **9** (3 molar equiv.) titrated into iron(III) nitrate.

Molecular Dynamics Data

Table S19: Average heavy-atom RMSD and standard deviation (Å) of the substrate over the total simulation time with the trajectory aligned based on the protein backbone.

Substrate	RMSD
Dopamine	2.752±0.611
**10**	5.329±2.378
**2**	5.407±0.839
**3**	5.021±1.765
**4**	4.137±0.805
**5**	3.825±0.697
**S-6**	6.445±2.349
**R-6**	3.606±0.652
**S-7**	4.456±1.126
**R-7**	4.938±1.234
**S-8**	5.447±1.172
**R-8**	3.938±0.880

Table S20: Frequency of the total simulation time in which residues are within 4 Å of the substrates. Only contacts that are formed for >30% of the total simulation time are shown.

	Dopamine	10	2	3	4	5	S-6	R-6	S-7	R-7	S-8	R-8
**PHE76**	90%	-	67%	66%	57%	78%	64%	86%	-	-	98%	73%
**ALA77**	-	-	-	-	-	-	-	-	-	-	49%	-
**ASP79**	88%	-	53%	88%	42%	33%	41%	82%	45%	74%	84%	71%
**LEU80**	-	-	-	49%	-	-	-	-	35%	-	-	-
**ALA81**	-	-	-	84%	-	-	-	-	-	-	-	-
**TRP84**	-	-	-	44%	-	-	-	-	-	-	-	-
**ARG85**	-	-	-	61%	-	-	-	-	-	-	-	-
**ILE148**	-	-	-	-	-	39%	-	-	-	-	-	-
**SER149**	100%	97%	60%	-	93%	99%	99%	100%	90%	100%	100%	100%
**LEU150**	-	-	-	-	-	-	-	-	33%	-	-	-
**VAL152**	89%	67%	72%	69%	75%	89%	72%	93%	81%	82%	92%	79%
**GLY153**	97%	70%	-	-	62%	32%	95%	94%	77%	97%	98%	97%
**PHE155**	-	-	-	47%	-	-	-	-	-	-	-	-
**TYR156**	100%	77%	69%	97%	95%	87%	98%	100%	97%	100%	100%	100%
**ASN157**	-	37%	-	-	40%	-	45%	-	44%	42%	-	-
**ILE160**	-	-	-	-	-	-	-	-	32%	-	-	-
**LEU251**	-	-	-	-	-	-	30%	-	-	-	-	-
**PHE320**	89%	44%	87%	97%	-	67%	40%	80%	45%	37%	86%	82%
**SER321**	-	-	36%	31%	33%	33%	35%	-	-	-	67%	50%
**LEU322**	-	-	32%	-	-	-	-	-	-	-	-	-
**GLY323**	71%	45%	72%	33%	35%	63%	48%	62%	-	-	87%	70%
**VAL324**	-	34%	-	-	-	-	-	-	-	-	-	-
**PHE326**	90%	43%	95%	88%	73%	90%	75%	97%	65%	94%	99%	96%
**VAL328**	65%	35%	67%	-	59%	70%	52%	74%	37%	77%	72%	66%
**GLY419**	-	-	-	-	-	-	-	-	31%	-	-	-
**ASP421**	-	-	37%	-	-	-	-	-	-	-	-	-
**SER422**	94%	67%	84%	48%	71%	69%	65%	85%	95%	92%	93%	90%
**ALA423**	91%	90%	-	-	96%	70%	90%	88%	90%	97%	90%	94%
**GLY425**			44%	-	-	-	-	-	-	-	-	-
**GLY426**	97%	74%	65%	-	75%	93%	87%	96%	88%	87%	95%	92%
**MET427**	96%	97%	-	-	98%	91%	98%	95%	82%	93%	96%	99%
**VAL430**	30%	55%	-	-	-	36%	64%	85%	48%	70%	49%	78%
**ILE452**	-	-	-	-	35%	-	36%	30%	-	-	-	-
**THR456**	-	45%	-	-	47%	-	50%	33%	39%	33%	-	31%
**ASP476**	-	-	-	41%	-	-	-	-	-	-	-	-
**Na+**	100%	-	48%	-	-	-	56%	93%	-	-	32%	85%

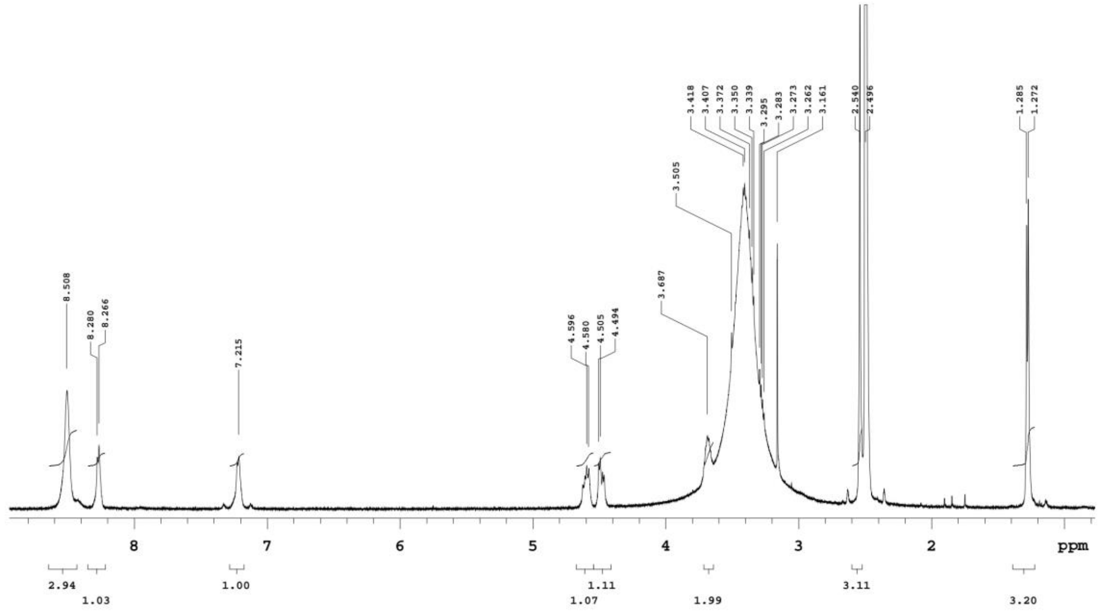

Figure S17: ^{1}H NMR spectrum of **1** (DMSO-d_6). Note that the spectra of **2** and **3** are identical.

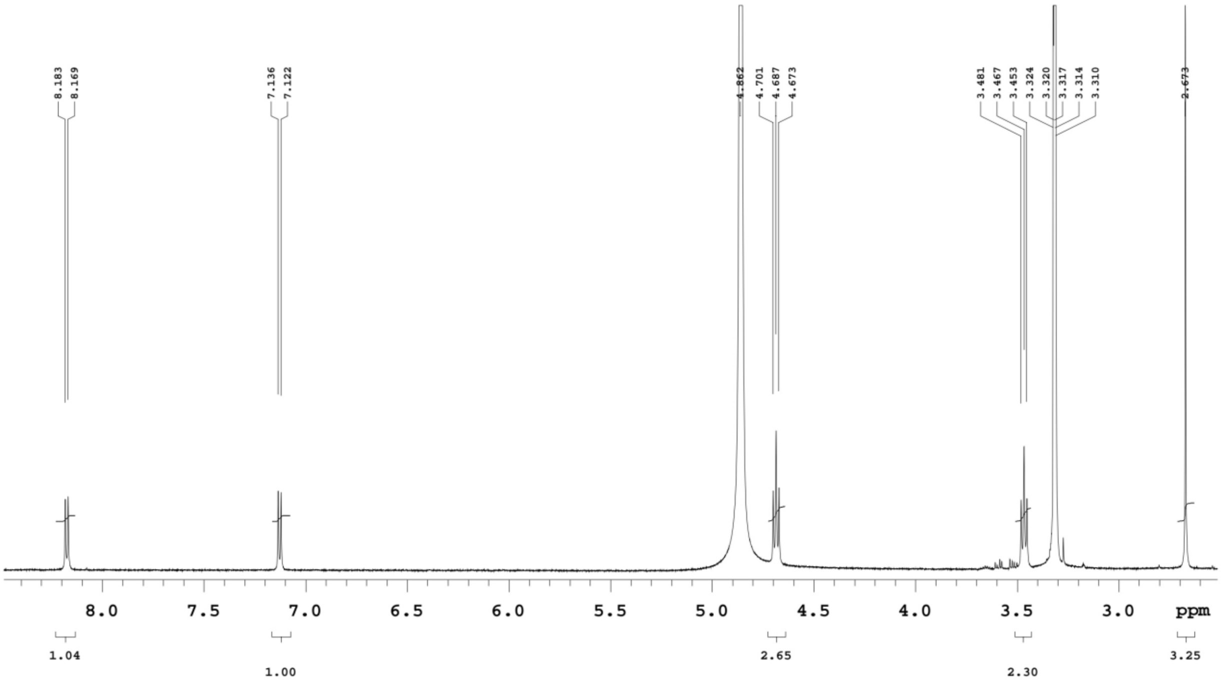

Figure S18: ^{1}H NMR spectrum of **4** (DMSO-d_6).

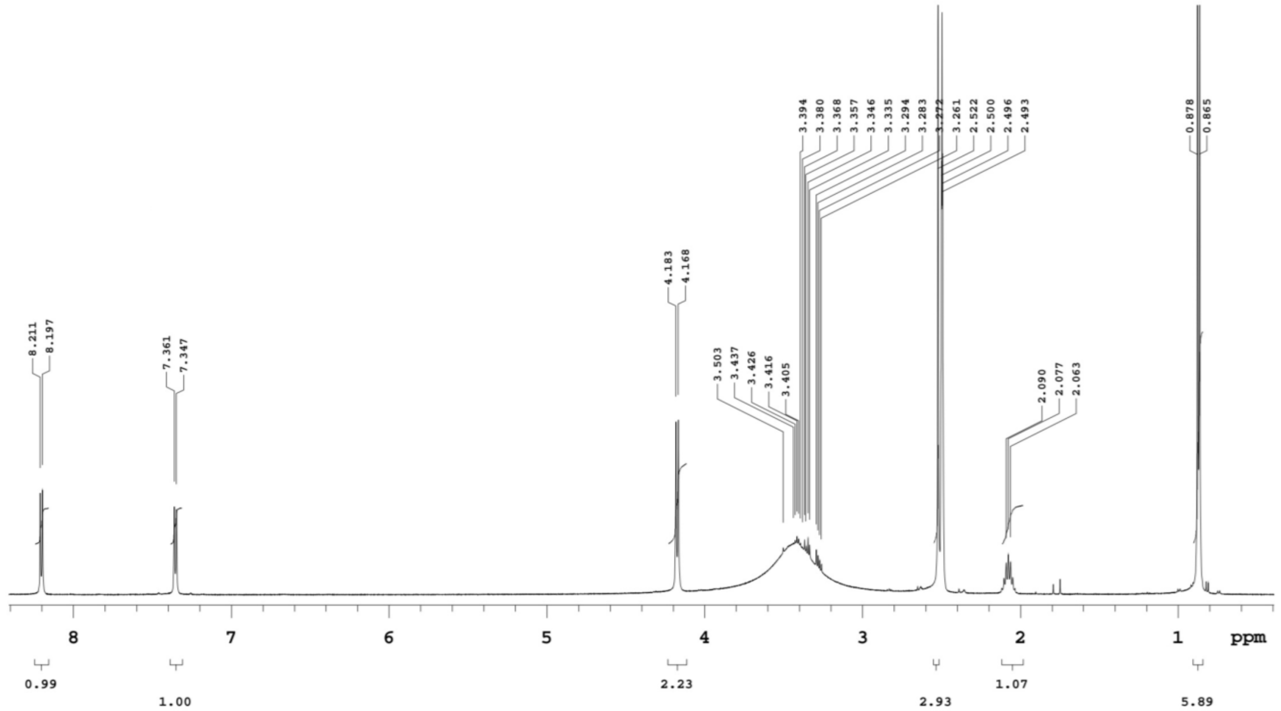

Figure S19: ^{1}H NMR spectrum of **5** (DMSO-d_6).

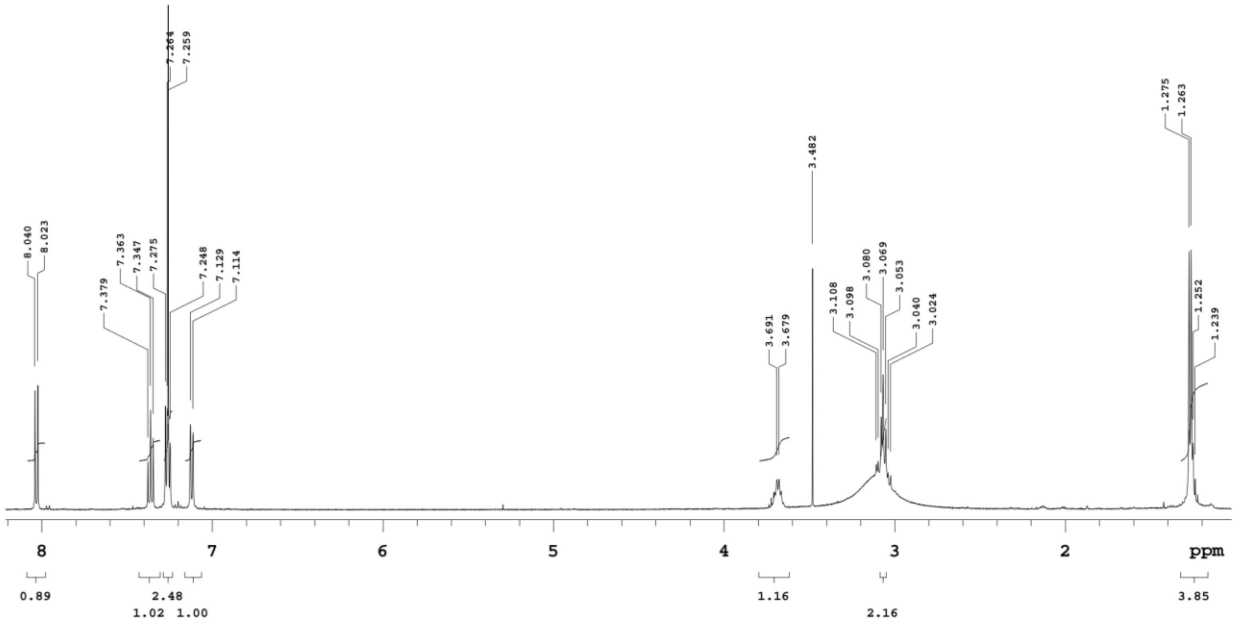

Figure S20: ^{1}H NMR spectrum of **6** ($CDCl_3$).

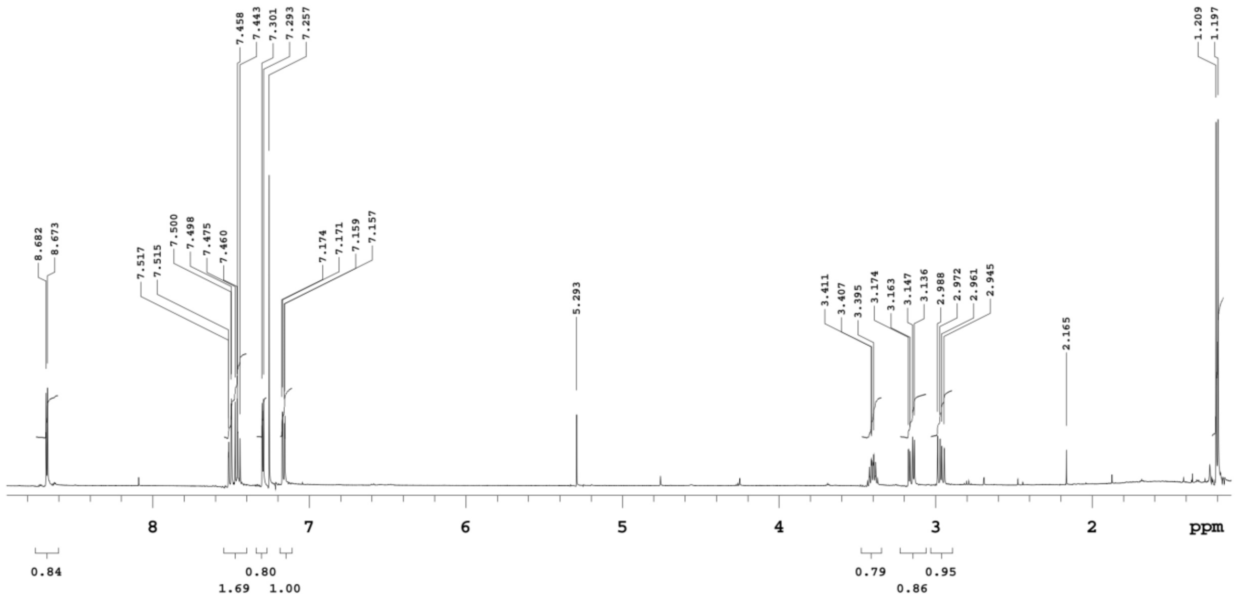

Figure S21: ^{1}H NMR spectrum of **7** ($CDCl_3$).

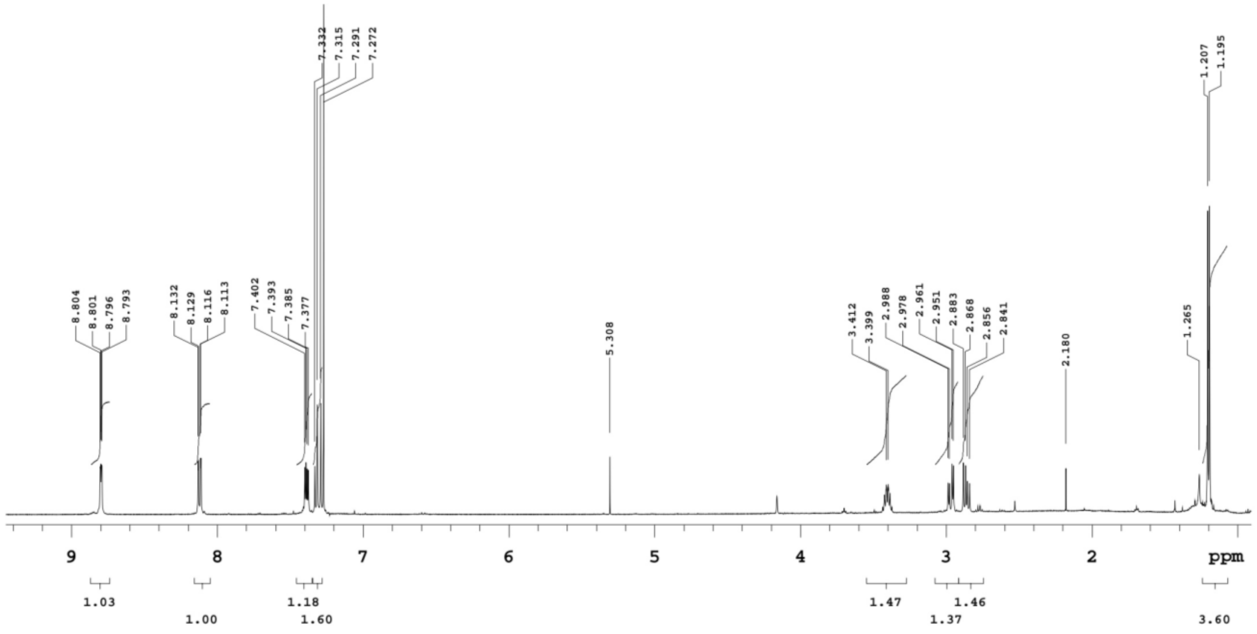

Figure S22: ^{1}H NMR spectrum of **8** ($CDCl_3$).

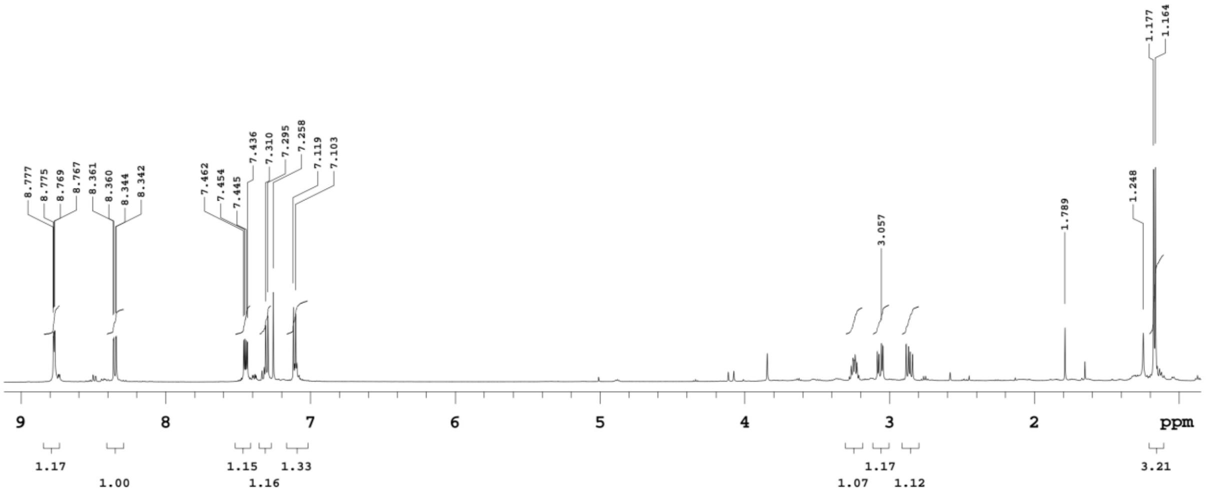

Figure S23: ^{1}H NMR spectrum of **9** ($CDCl_3$).
